# Conditional overexpression of PPARα in intestinal epithelium diminishes GIP enteroendocrine cells and circulating hormone levels

**DOI:** 10.1016/j.molmet.2026.102429

**Published:** 2026-08-13

**Authors:** Jacob J. Socha, Swathi Pavithran, Courtney A. Burger, Rebekah Karns, Avelina W. Lee, Richard Lang, Maria E. Moreno-Fernandez, Stephen W. Standage, Kelli L. VanDussen

**Affiliations:** 1 Division of Gastroenterology, Hepatology, and Nutrition, Cincinnati Children's Hospital Medical Center, Cincinnati, OH, USA; 2 Division of Pediatric Ophthalmology, Cincinnati Children's Hospital Medical Center, Cincinnati, OH, USA; 3 Abrahamson Pediatric Eye Institute, Cincinnati Children's Hospital Medical Center, Cincinnati, OH, USA; 4 Science of Light Center, Cincinnati Children's Hospital Medical Center, Cincinnati, OH, USA; 5 Department of Ophthalmology, College of Medicine, University of Cincinnati, Cincinnati, OH, USA; 6 Division of Developmental Biology, Cincinnati Children's Hospital Medical Center, Cincinnati, OH, USA; 7 Division of Critical Care Medicine, Cincinnati Children's Hospital Medical Center, Cincinnati, OH, USA; 8 Center for Stem Cell and Organoid Medicine, Cincinnati Children's Hospital Medical Center, Cincinnati, OH, USA; 9 Department of Pediatrics, University of Cincinnati College of Medicine and Cincinnati Children's, Hospital Medical Center, Cincinnati, OH, USA

**Keywords:** Peroxisome proliferator-activated receptor alpha, Intestine, Epithelium, GIP, Incretin, Satiety

## Abstract

Agonists of the lipid-sensing PPAR nuclear receptors, including PPARα, are being explored as therapies for metabolic disorders due to their roles in metabolic and anti-inflammatory processes. PPARα transcriptional programs have tissue-dependent features, yet there is a lack of genetic tools to study tissue-specific signaling activation without the addition of a systemic agonist. We aimed to investigate intestinal epithelial cell (IEC)-specific roles of PPARα signaling using a novel transgenic mouse that enables spatial and temporal control of *Ppara* overexpression. CAG-*Ppara*,-*EGFP* mice were bred to *Villin-CreERT2* to establish the IEC-*Ppara* mouse, which was compared to littermate controls 2 weeks after tamoxifen exposure. IEC-*Ppara* mice had increased *Ppara* mRNA and PPARα protein in the intestinal epithelium. Transcriptional analysis of intestinal tissue from IEC-*Ppara* mice showed upregulation of PPARα target genes and functional enrichment for fatty acid catabolic processes. As expected, the enterocytes of IEC-*Ppara* mice were primed to absorb lipids following oral administration of an olive oil bolus. Unexpectedly, the enteroendocrine hormone *Gip* was among the most downregulated genes. GIP-positive cells were reduced in the intestines of IEC-*Ppara* mice and in mice treated with PPARα agonist WY-14643. Circulating GIP hormone was reduced in IEC-*Ppara* mice. GLP-1-positive cells and hormone were unchanged. Consistent with reduced GIP function, IEC-*Ppara* mice consumed more food. These findings reveal PPARα as a regulator of GIP and support a new framework in which PPARα signaling influences systemic energy balance via a gut hormone axis. This study could have future impact on understanding responses to therapies targeting PPAR or incretin signaling.

## HIGHLIGHTS

• A novel mouse strain for conditional, genetic overexpression of *Ppara* was generated.
• IEC-*Ppara* mice specifically overexpressed *Ppara* in the intestinal epithelium.
• IEC-*Ppara* mice showed enhanced intestinal PPARα signaling and lipid uptake capacity.
• PPARα activation in the intestinal epithelium decreased GIP enteroendocrine cells.
• IEC-*Ppara* mice had lower GIP, but not GLP-1, hormone and ate more chow.

## Graphical abstract

**Figure:**
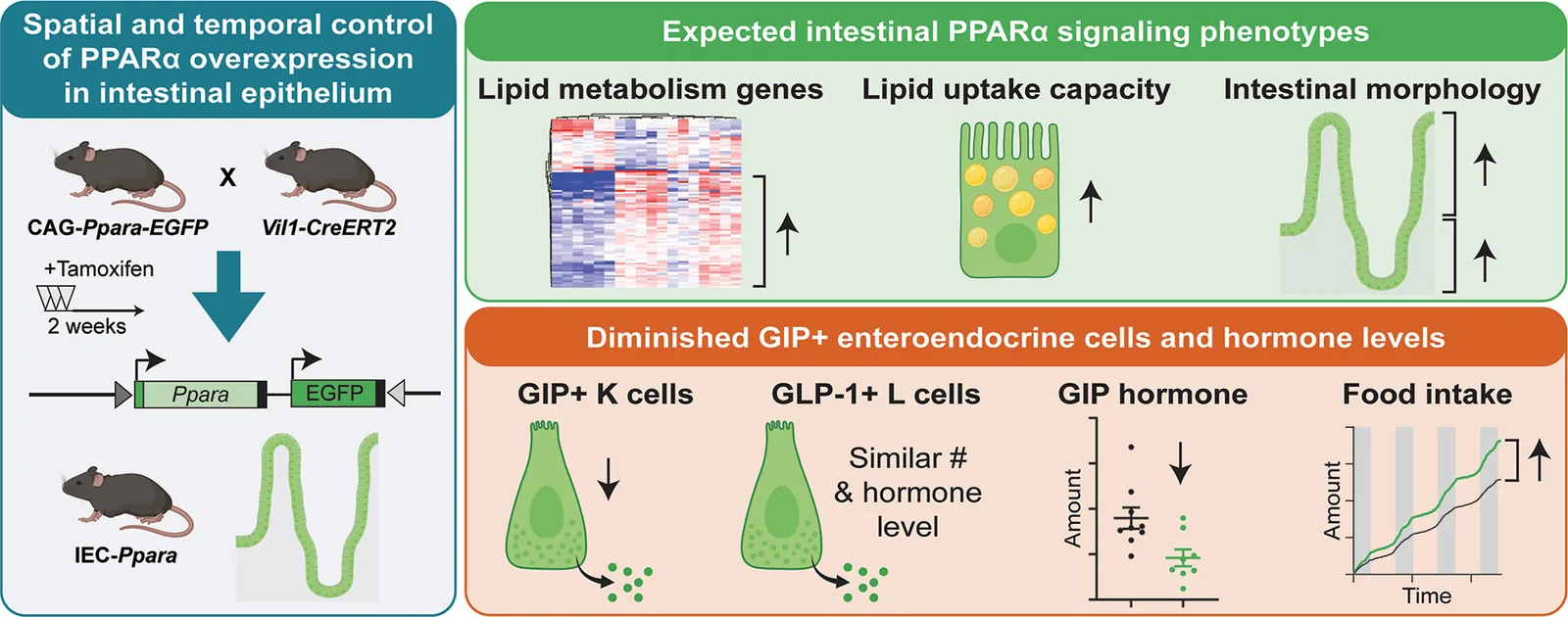

## 1 Introduction

The small intestine, as the primary site of nutrient absorption, integrates signals from dietary components and gut microbiota to coordinate systemic energy balance, metabolism, and glucose homeostasis alongside other tissues such as the pancreas, liver, and brain [1,2]. Nuclear receptors represent one mechanism by which the intestinal epithelium senses and respond to dietary signals via transcriptional regulation of metabolic programs [3]. The Peroxisome Proliferator Activated Receptors (PPARs), including PPARα, PPARδ, and PPARγ, are lipid-responsive nuclear hormone receptors. PPAR transcriptional activity is highly context-dependent, shaped by the availability and affinity of their lipid ligands and co-regulators as well as by cell and tissue types [4]. Whereas PPARγ and PPARδ are established regulators of glucose homeostasis, acting in white adipose tissue, skeletal muscle, and liver to improve insulin sensitivity, PPARα has been primarily associated with hepatic lipid metabolism, where it promotes fatty acid transport, β-oxidation, and ketogenesis [[5], [6], [7]]. Due to their roles in lipid metabolism and anti-inflammatory processes, selective PPAR agonists are being actively pursued as therapies in clinical and pre-clinical studies for metabolic diseases [[8], [9], [10], [11], [12], [13]]. Thus, it is critical to improve our understanding of the context-specific and tissue-specific effects of PPAR signaling.

PPARα is highly expressed in the small intestinal epithelium [14,15], but its roles in the intestine are relatively understudied compared to liver, adipose, and muscle tissues. WY-14643 is a PPARα agonist and the most widely used experimental approach to activate PPARα signaling in rodent models. Short-term WY-14643 treatment of wildtype, but not *Ppara* null, mice induced genes functionally related to fatty acid metabolism, triacylglyceride metabolism, sterol biosynthesis, and bile acid metabolism in the small intestine [14]. Conversely, systemic PPARα antagonism or mice with conditional *Ppara* loss-of-function in the intestinal epithelium (*Ppara*^ΔIEC^) showed dramatically diminished lipid uptake by small intestinal enterocytes [16,17]. In a 12-week high-fat diet study, diminished lipid uptake in the intestines of *Ppara*^ΔIEC^ mice associated with reduced hepatic lipid accumulation, reduced weight gain, and attenuated features of metabolic dysfunction-associated steatohepatitis (MASH) [17], highlighting one example of inter-organ crosstalk mediated by PPARα signaling. WY-14643 administration also lengthened the small intestinal villi and enhanced stem cell activity [14,18], whereas *Ppara*^ΔIEC^ mice had reduced villus height [16,17]. In sum, our current understanding is that PPARα signaling regulates lipid metabolism, tissue morphology, and stem cell activity in the intestinal epithelium.

However, it is important to consider that the systemic actions of PPARα signaling could confound the interpretation of its tissue-specific roles. It is well-described that WY-14643 reduces food intake, an effect mediated via hepatocytes [7]. This is particularly relevant to the intestine, as food intake itself influences intestinal stem cell activity and tissue morphology [16,[19], [20], [21], [22]]. High-fat diets are also frequently used to model PPARα signaling activation [17,18] and produce a plethora of direct and indirect effects on intestinal function [23]. Thus, it is not always clear if the intestinal phenotypes observed with WY-14643 or high-fat diet are directly or indirectly due to PPARα signaling in the intestine. The recent development of conditional *Ppara* loss-of-function mouse models has been an important advance for the dissection of tissue-specific roles of PPARα signaling. Yet *Ppara*^ΔIEC^ mice are typically studied in combination with a PPARα agonist (WY-14643 or high-fat diet), and thus it is still possible that observed phenotypes are indirect. Finally, some studies have reported PPARα-independent effects of WY-14643 [[24], [25], [26]].

A genetic mouse strain enabling the spatial and temporal control of *Ppara* overexpression would provide a complementary *in vivo* approach to test tissue-specific roles of PPARα signaling. The feasibility of such a genetic approach is supported by studies in which muscle-specific gene promoters were successfully used to produce transgenic mice with tissue-specific *Ppara* overexpression [27,28]. Here, we generated a novel mouse strain via targeted transgene insertion that yields conditional *Ppara* overexpression upon Cre-mediated genetic inversion. We bred these mice to *Villin-CreERT2* [29] to investigate the roles of PPARα signaling in the intestinal epithelium of adult mice. As expected, two weeks after induction of genetic recombination, we found that intestinal epithelial-intrinsic PPARα overexpression was sufficient to rapidly remodel epithelial transcriptional programs toward fatty acid uptake and oxidation, functionally enhance enterocyte lipid absorption capacity, and increase villus and crypt heights. Unexpectedly, we revealed a previously unrecognized role for intestinal PPARα signaling in the regulation of the gut incretin hormone GIP. This exciting finding supports a novel, tissue-specific mechanism by which intestinal PPARα signaling can influence systemic energy balance.

## 2 Methods

### 2.1 Mice

The *C5*7BL*/6J-Col1a1*^*em1(CAG-Ppara,-EGFP)Kvd*^*/Kvd* mouse strain was generated by the Cincinnati Children's Hospital Medical Center Transgenic Animal and Genome Editing Facility. This strain conditionally expresses a FLAG-*Ppara*-IRES-*EGFP* transgene from the safe harbor at the 3′ UTR of the *Col1a1* locus. In brief, a donor vector was constructed in which a N-terminal FLAG tagged *Ppara*-IRES-*EGFP*-SV40 polyA sequence in the reverse orientation was flanked by *Lox66* and *Lox71* sites, which allow only one-way inversion upon expression of Cre [30,31]. The cassette was put under control of the CAG promoter on the 5′-side and protected from unwanted expression by an insulator on the 3′-side. The entire cassette was flanked by 2.5 kb and 2.9 kb at the 5′ and 3′ ends, respectively, of homologous arms of the *Col1a1* safe harbor locus. The vector was targeted to the *Col1a1* safe harbor locus with the aid of a Cas9-gRNA that cleaves the target site. The pre-assembled Cas9-gRNA complex along with the plasmid donor was microinjected into pronuclei of fertilized C57BL6/J eggs, which were then implanted into C57BL6/J pseudopregnant females. The oligonucleotide sequences to verify correct insertion of the transgene and for PCR genotyping are listed in Table S1. PCR genotyping to detect the CAG-*Ppara*,-*EGFP* transgene used internal oligos P1 and P2 and external oligos PparaTGLHA and PparaTGRHA with thermocycler conditions denaturing at 95 °C for 30s, annealing at 60 °C for 30s, and extension at 72 °C for 45s for 35 cycles. *Ppara* floxed (*Ppara*^flox/flox^; targeting Exon 5) mice were provided by Dr. Standage. These mice were generated from *Ppara* floxed embryonic stem (ES) cells (para^tm1a(EUCOMM)Wtsi^, Clone EPD0157_5_A06) purchased from the European Conditional Mouse Mutagenesis (EUCOMM) consortium. ES cells were micro-injected into blastocysts harvested from albino B6(Cg)-Tyr^c−2J^/J mice (Jax #000058) and then then implanted in pseudo-pregnant dams. Chimeric pups were bred against albino mates when mature to establish the F1 generation. These were bred to homozygosity at the transgenic *Ppara* locus and then crossed with a FLPase expressing mouse (B6·129S4-Gt (ROSA)26Sor^tm2(FLP^∗^)Sor^/J, Jax #012930) to remove the targeting vector/gene trap. Progeny from these crosses bearing the successfully edited transgene allele were backcrossed against wild type C57BL/6 J (Jax #00064) for 10 generations achieve syngeneic status and then bred to homozygosity again at the *Ppara* locus. To drive transgene expression in intestinal epithelium, CAG-*Ppara*,-*EGFP* mice or *Ppara*^flox/flox^ mice were crossed with *Villin-CreERT2* mice (The Jackson Laboratory, #020282) [29].

All animal studies were performed according to protocols approved by the Cincinnati Children's Hospital Medical Center Institutional Animal Care and Use Committee. Experiments used littermate male and female mice on the C57BL6/J strain background at 8–13 weeks of age, unless otherwise stated. To induce Cre-mediated genetic recombination, mice were administered tamoxifen (100 mg/kg) or an equivalent volume of corn oil as a negative control via intraperitoneal injection daily for 3 consecutive days. Mice were analyzed 2 weeks after the final tamoxifen injection. Mice were maintained in a specific pathogen-free barrier facility with a 14-hr light cycle, temperature of (22.2 °C), and humidity (30%–70%) with *ad libitum* access to water and chow (Teklad #2919; 3.3 kcal/g with 23% calories from protein, 22% calories from fat, and 55% calories from carbohydrate), unless stated otherwise. For experiments involving administration of WY-14643, mice were either fed irradiated control diet with 10% kcal fat (Research Diets, D12450Hi) or the same control diet admixed with 0.1% WY-14643 (Research Diets, D21060407i) for 1 week. WY-14643 was purchased from Cayman Chemical.

### 2.2 Tissue collection and histology

For small intestinal tissue collection, the small intestine was dissected and divided into four segments of equal length. From proximal to distal, they were labeled A, B, C, and D. Duodenum, jejunum, and ileum were considered to be segments A, C, and D, respectively. Each segment was further subdivided into four pieces of equal length (1–4). For each region, formalin-fixed, paraffin-embedded (FFPE) tissues were prepared from piece 1; paraformaldehyde-fixed or unfixed frozen tissues were prepared from piece 2, and RNA was isolated from piece 4. FFPE tissues were fixed in 10% formalin overnight at 4 °C, rinsed in 70% ethanol 3 times for 10 min, and blocked in 2% agar prior to paraffin processing and embedding. Fixed-frozen tissues were fixed in 4% paraformaldehyde (PFA) in PBS overnight at 4 °C, rinsed in 20% sucrose 2 times for 3 h, and blocked in OCT prior to freezing and storage at −80 °C. For liver collection, whole livers were dissected out and weighed. The left lobe was cut in half, and the two halves were longitudinally cut into 3 pieces that were either fixed in 10% formalin or 4% paraformaldehyde at 4 °C while rotating for ∼2 days. Then, the fixative was removed, and tissues were rinsed with 1X PBS. For FFPE processing, livers were rinsed again with 1X PBS and set on a rotator at room temperature in 1X PBS for 20 min to equilibrate. They were then blocked in 2% agar prior to paraffin processing and embedding. For fixed-frozen blocks, livers were rinsed in 20% sucrose overnight at 4 °C and then blocked in OCT prior to freezing and storage at −80 °C. The medial lobe of the liver was used for RNA isolation and flash freezing in liquid nitrogen. For fat pad analysis, inguinal white adipose tissue (iWAT), perirenal white adipose tissue (pWAT), gonadal white adipose tissue (gWAT), and brown adipose tissue from the interscapular region (BAT) were dissected from fasted mice, weighed, placed in a tissue cassette, and then fixed in 10% formalin for ∼2 days. Tissues were rinsed in 70% ethanol twice and then processed for paraffin embedding.

### 2.3 Immunofluorescence, RNAscope, and immunohistochemistry

Endogenous transgene-derived EGFP expression used Pfa-fixed frozen histology cryosections (12-μm) stained with DAPI to visualize nuclei. Representative fluorescent images were acquired with a Nikon NiE microscope, Digital Sight 10 camera, and Nikon NIS-Elements software. Pfa-fixed frozen sections (12-μm) were used for RNAscope according to the manufacturer's protocol using the RNAScope 2.5 HD Reagent Kit-RED kit (ACD Biotechne, #322350) and murine *Ppara* probe (ACD Biotechne, Mm-Ppara #454051). For immunohistochemistry, FFPE histological sections (4–5-μm) were deparaffinized in xylene, rinsed in isopropanol, rehydrated in water, boiled in Trilogy (Millipore Sigma) or Citrate buffer (Millipore Sigma) antigen retrieval solution for 20–30 min, and then rinsed in PBS. Endogenous peroxide activity was exhausted by incubating slides in 3% hydrogen peroxide in methanol for 45 min followed by a PBS rinse. Blocking was performed with 0.1% Triton X-100 and 1% BSA in PBS for 30 min followed by incubation with primary antibodies diluted in blocking buffer. Primary antibodies included PPARα (Abcam, #ab245119; 1:100 dilution), GIP (Abbiotec, #250231; 1:500 dilution), ChgA (Abcam, #ab254557; 1:500 dilution), and GLP-1 (BioVision, #3104–100; 1:200 dilution). Slides were next incubated with biotin-conjugated secondary antibodies (Thermo Fisher Scientific) followed by visualization with the Vectastain Elite ABC and DAB (3,3′-diaminobenzidine) substrate kits (Vector Laboratories). Sections were counterstained in hematoxylin, dehydrated via a series of ethanol and xylene, followed by mounting of coverslips with Permount (ThermoFisher Scientific). Representative brightfield images were acquired with an Olympus BX53 microscope equipped with UPlanSApo 4X/0.16, 10X/0.40, 20X/0.75, 40X/0.95 Oil, and 100X/1.40 Oil objective lenses, an Olympus UC90 camera, and CellSens software. Adobe Photoshop was used to uniformly adjust brightness and contrast and to crop images. To reduce the potential for bias, image analysis was performed using randomly coded samples stripped of group identifiers by researchers who were blinded to the group assignment.

### 2.4 RNA isolation and qRT–PCR

RNA was isolated and DNase-treated using a NucleoSpin RNA kit (Takara), and cDNA reactions used 1 μg of RNA and iScript Reverse Transcriptase Supermix (Biorad), both according to the manufacturers’ protocols. Quantitative PCR reactions were performed in technical duplicates and used TB Green Advantage qPCR Premix (Takara) and the primers listed in Table S2. Primer efficiencies were previously validated using a serial dilution series and standard curve analysis. Relative gene expression levels were normalized to a housekeeper gene, whose expression remained consistent, using the delta–delta Ct method.

### 2.5 RNA-seq profiling

RNA-sequencing was performed by the Genome Technology Access Center (GTAC) at Washington University in St. Louis. Library preparation was performed with 5 to 10 μg of total RNA with an Agilent Bioanalyzer RIN score greater than 8.0. Ribosomal RNA was removed by poly-A selection using Oligo-dT beads (mRNA Direct kit, Life Technologies). mRNA was then fragmented in reverse transcriptase buffer by heating to 94° for 8 min and reverse transcribed to cDNA using SuperScript III RT enzyme (Life Technologies) and random hexamers. A second strand reaction was performed to yield ds-cDNA. cDNA was blunt ended, had an A base added to the 3′ ends, and then had Illumina sequencing adapters ligated to the ends. Ligated fragments were amplified for 12–15 cycles using primers incorporating unique dual index tags. Fragments were sequenced on an Illumina NovaSeq-6000 using paired end reads extending 150 bases. Base calls and demultiplexing were performed with Illumina's bcl2fastq software with a maximum of one mismatch in the indexing read. RNA-seq reads were aligned to the Ensembl release 101 primary assembly with STAR version 2.7.9a1 [32]. Gene counts were derived from the number of uniquely aligned unambiguous reads by Subread:featureCount version 2.0.32 [33]. Sequencing performance was assessed for the total number of aligned reads, total number of uniquely aligned reads, and features detected. The ribosomal fraction, known junction saturation, and read distribution over known gene models were quantified with RSeQC version 4.04 [34]. Genes with CPM >3 in at least 4 samples were included in downstream analyses (n = 12,750 genes). From the protein-coding genes (n = 11,804), we identified differentially expressed genes (*P* < 0.001) based on the following genotype comparisons: IEC-*Ppara* heterozygous vs. control (n = 22), IEC-*Ppara* homozygous vs. control (n = 42 genes), and IEC-*Ppara* homozygous vs. heterozygous (n = 15). In total, 95 genes were included in downstream analyses. Unsupervised hierarchical clustering was performed using log-transformed CPM data baselined to the median of all samples. ToppGene and ToppCluster platforms [35,36] and Enrichr [37] were used for functional annotation enrichment analyses and Cytoscape. v3.0.2 [38] was used for visualization. Sequencing data have been deposited at NCBI GEO with accession GSE336031.

### 2.6 Serum hormone and lipid analyses

Blood was collected via cardiac puncture into Serum Separator Tubes (ThermoFisher Scientific) and prepared according to manufacturer's directions. Analysis of serum triglycerides, cholesterol, and HDL from serum of *ad libitum* fed mice was performed by IDEXX Bioanalytics (North Grafton, MA) using the Rodent Lipid Panel. For gut hormone analysis, mice were fasted overnight for 11 h. In the morning, a portion of the mice were administered 200 μL of original flavor Ensure nutritional shake via oral gavage 30 min prior to blood collection. DPP IV inhibitor (Millipore Sigma, DPP4-M; final concentration 40 μM) and Protease Inhibitor Cocktail (Millipore Sigma, P2714-1BTL; final concentration of 1X) were immediately mixed with blood samples upon collection. Gut hormone protein concentrations were determined using a custom MILLIPLEX Mouse Metabolic Hormone Magnetic Bead Panel multiplex assay (Millipore Sigma) by the Research Flow Cytometry Facility at Cincinnati Children's Hospital Medical Center. Technical duplicates were run and averaged. GLP-1 hormone concentrations were determined with the Mercodia Total GLP-1 NL-ELISA kit (Mercodia Inc, #10-1278-01) according to the manufacturer's instructions. If the concentration of a sample was below the lower limit of quantification (LLOQ), then its value was set at LLOQ/2. If >50% of the samples were below the LLOQ in both genotypes, the hormone was removed from further analysis. Outliers identified in GraphPad using the ROUT method with Q = 1% were removed from further analysis.

### 2.7 Glucose tolerance test

Glucose tolerance tests were performed as previously described [39]. In brief, mice were fasted overnight for 11 h. In the morning, a 10% dextrose solution was prepared by diluting dextrose anhydrous (Fisher Scientific, #BP350) in 0.9% Sodium Chloride. Mice were then administered intraperitoneal injections of 10 μL of 10% dextrose solution per gram of body weight. Blood glucose levels were measured via glucometer (McKesson) at 0, 20, 40, 60, 90, and 120 min post-injection.

### 2.8 OilRedO staining and quantification

To assess intestinal lipid uptake, mice were treated with tamoxifen as previously described. Prior to the tissue harvest, mice were fasted overnight and then administered a 250 μL bolus of olive oil by oral gavage 2-hr prior to tissue collection. Unfixed intestinal tissue was collected and immediately frozen in OCT. To assess liver lipid content, the left lobe of the liver was dissected into thirds and fixed in 4% Pfa for at least 24 h. Fixed liver was then blocked in OCT. Intestinal and liver tissue cryosections (12-μm) were dried at 60 °C for 1 h, fixed in 4% Pfa for 5 min, and then stained for OilRedO (Newcomer Supply) according to the manufacturer's protocol. Quantification of representative brightfield images used ImageJ [40] and a previously developed macro for OilRedO image analysis [41], with the only modification being that the saturation minimum was set to 50, to identify the proportion of the image that contained positive OilRedO staining. This value was normalized to the proportion of image that contained intestinal epithelium or liver tissue. Image analysis was performed in a single-blind fashion.

### 2.9 Indirect calorimetry

Indirect calorimetry experiments and analysis were performed according to a recently published consensus guide [42]. One week after the final tamoxifen injection, mice were weighed and singly housed in metabolic cages (Promethion, SABLE Systems International) with corn-cob bedding (1/4” Bed-o’ Cobs, The Andersons’). The temperature was set to 22 °C with a 14 L:10D light/dark cycle. No mid-study interventions were performed. A body mass hopper enclosure was present in the cages for enrichment. Three separate runs were completed over a 6-month period, and the results were combined. Following a 48-hr acclimatization period, food intake, water intake, oxygen consumption (VO_2_), carbon dioxide emission (VCO_2_), and directed locomotion were measured over ∼3.5 days (83.5-hr period). Mice were monitored and removed from the study if they exhibited food grinding behavior or went into torpor. Raw data were processed using MacroInterpreter software (SABLE Systems International). Directed locomotion was measured as the sum of distance traveled within the beam break system where the animal was traveling at 1 cm/s or greater. One mouse was removed from locomotion data due to the laser system malfunctioning. The RER was calculated by dividing carbon dioxide emission by oxygen consumption (VCO_2_/VO_2_). Energy expenditure was calculated using the Weir equation. Energy balance was calculated by taking the food intake in kcal and subtracting energy expenditure. Data was primarily analyzed in CalR v2 [43] and visualized with GraphPad Prism. Analysis of covariance (ANCOVA) using body weight as the covariate [44] was performed in CalR for food intake, VCO_2_, VO_2_, RER, and energy expenditure.

### 2.10 Bomb calorimetry

Mice were individually housed in cages with fresh ISO-Pad bedding (Teklad). Upon individual housing, mice were weighed along with food present in the food hopper. After 24 h, mice were weighed along with food remaining in food hopper. After another 24 h (48 h after the start of the single housing) mice were weighed along with food remaining in the food hopper. All fecal pellets present in the cage were then collected, taking care to separate from the bedding, weighed (wet weight), and stored at −80 °C until drying. To dry, samples were placed in a 60 °C oven for 48 h and then weighed (dry weight). Bomb calorimetry analysis was performed by The Metabolic Phenotyping Center at Weill Cornell Medicine.

### 2.11 Statistics

Normally distributed quantitative variables were described with means and standard error of the means. Appropriate 2-sided parametric or nonparametric statistical tests were applied after testing for normal distribution of data. Quantitative variables were compared by unpaired t tests for 2 groups or by 1-way ANOVA and Tukey's multiple comparisons posttest for more than 2 groups. Within graphs, either exact *P*-values are provided or ∗*P* < 0.05, ∗∗*P* < 0.01, ∗∗∗*P* < 0.001, ∗∗∗∗*P* < 0.0001, and ns (non-significant) indicate significance of comparisons. GraphPad Prism v9 was used to perform statistical analyses, unless otherwise indicated.

## 3 Results

### 3.1 IEC-*Ppara* mice produce inducible *Ppara* overexpression in the intestinal epithelium

**Fig. 1:**
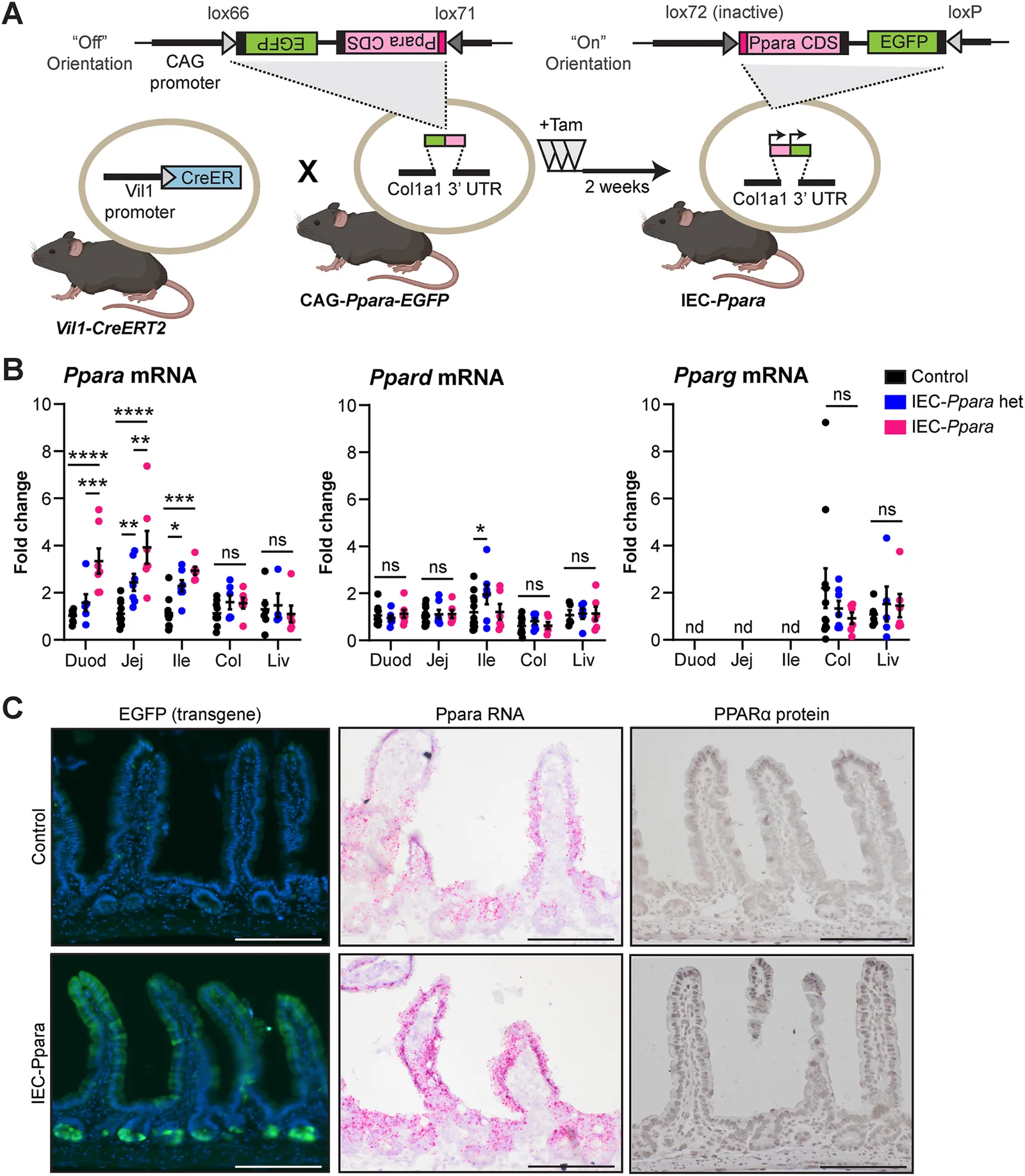
**IEC-*Ppara* mice yield tamoxifen-inducible, genetic overexpression of *Ppara* specifically in intestinal epithelium.** (**A**) Schematic of the novel CAG-*Ppara*,-*EGFP* mouse strain and the experimental strategy to yield tamoxifen-inducible genetic recombination in the intestinal epithelium. IEC-*Ppara* and control mice lacking Vil-CreERT2 were administered tamoxifen daily for 3 days and assessed 2 weeks later. (**B**) *Ppara*, *Ppard*, and *Pparg* gene expression determined by qRT-PCR in duodenum (Duod), jejunum (Jej), ileum (Ile), colon (Col), and liver (Liv) from control (black, n = 6–12), IEC*-Ppara* heterozygous (blue, n = 4–7), and IEC*-Ppara* homozygous (pink, n = 6–7) mice. ∗*P* < 0.05, ∗∗*P* < 0.01, ∗∗∗*P* < 0.001, and ∗∗∗∗*P* < 0.0001, ns (not significant) as determined by 2-way ANOVA and Tukey's multiple comparisons. Nd, indicates gene not consistently detected in the indicated tissue. (**C**) Representative microscopic images showing expression of endogenous EGFP (left), *Ppara* RNA determined by RNAscope (middle), and PPARα protein determined by immunohistochemistry (right) in control and IEC-*Ppara* homozygous ileal tissues. Scale bars, 100 μm.

We generated a novel mouse strain for conditional, genetic overexpression of CAG-*Ppara*,-*EGFP* from a genomic safe harbor site within the 3-UTR of the *Col1a1* locus (Figure 1A, Fig. S1A–B). Crossbreeding these mice yielded heterozygous or homozygous progeny, which could be distinguished by PCR genotyping (Fig. S1C). CAG-*Ppara*,-*EGFP* mice were crossed to *Villin-CreERT2* mice to enable tamoxifen-inducible *Ppara* overexpression specifically in the intestinal epithelium of adult mice. Heterozygous or homozygous CAG-Ppara,-*EGFP* mice expressing *Villin-CreERT2* (herein referred to as IEC-*Ppara*) were compared to those lacking Cre expression (herein referred to as controls) two weeks after tamoxifen administration (Fig. 1A). As expected, IEC-*Ppara* mice showed increased *Ppara* mRNA expression throughout the intestine vs. controls (Fig. 1B). Generally, *Ppara* mRNA expression tended to be higher in small intestinal tissues of IEC-*Ppara* homozygous (range: 2.9–3.9-fold vs. controls) than heterozygous (range: 1.6–2.4-fold vs. controls) mice. IEC-*Ppara* vs. control mice administered corn oil alone (vehicle) had similar *Ppara* mRNA levels (Fig. S1D). Confirming tissue specificity, *Ppara* mRNA levels in the liver were unchanged (Fig. 1B). Supporting a lack of gene compensation, the expression levels of the other PPAR family members, *Ppard* and *Pparg*, were also unaffected in IEC-*Ppara* intestine or liver tissues (Fig. 1B). We next assessed whether the transgene was appropriately expressed in intestinal epithelium. Transgene-driven EGFP expression was restricted to the intestinal epithelium in IEC-*Ppara* mice and was not observed in control mice (Figure 1C). Stronger EGFP expression was observed in crypt bases and upper villus epithelium. These locations contain long-lived Paneth cells and late-stage absorptive enterocytes, respectively, and thus presumably these cells had accumulated EGFP protein over time. We also observed increased *Ppara* mRNA and PPARα protein expression in the intestinal epithelium of IEC-*Ppara* mice vs. controls (Fig. 1C). Overall, these data demonstrate that the novel IEC-*Ppara* mouse strain faithfully enables conditional, genetic overexpression of *Ppara* in our target tissue, the intestinal epithelium.

### 3.2 IEC-*Ppara* mice show elevated intestinal PPARα signaling activity

**Fig. 2:**
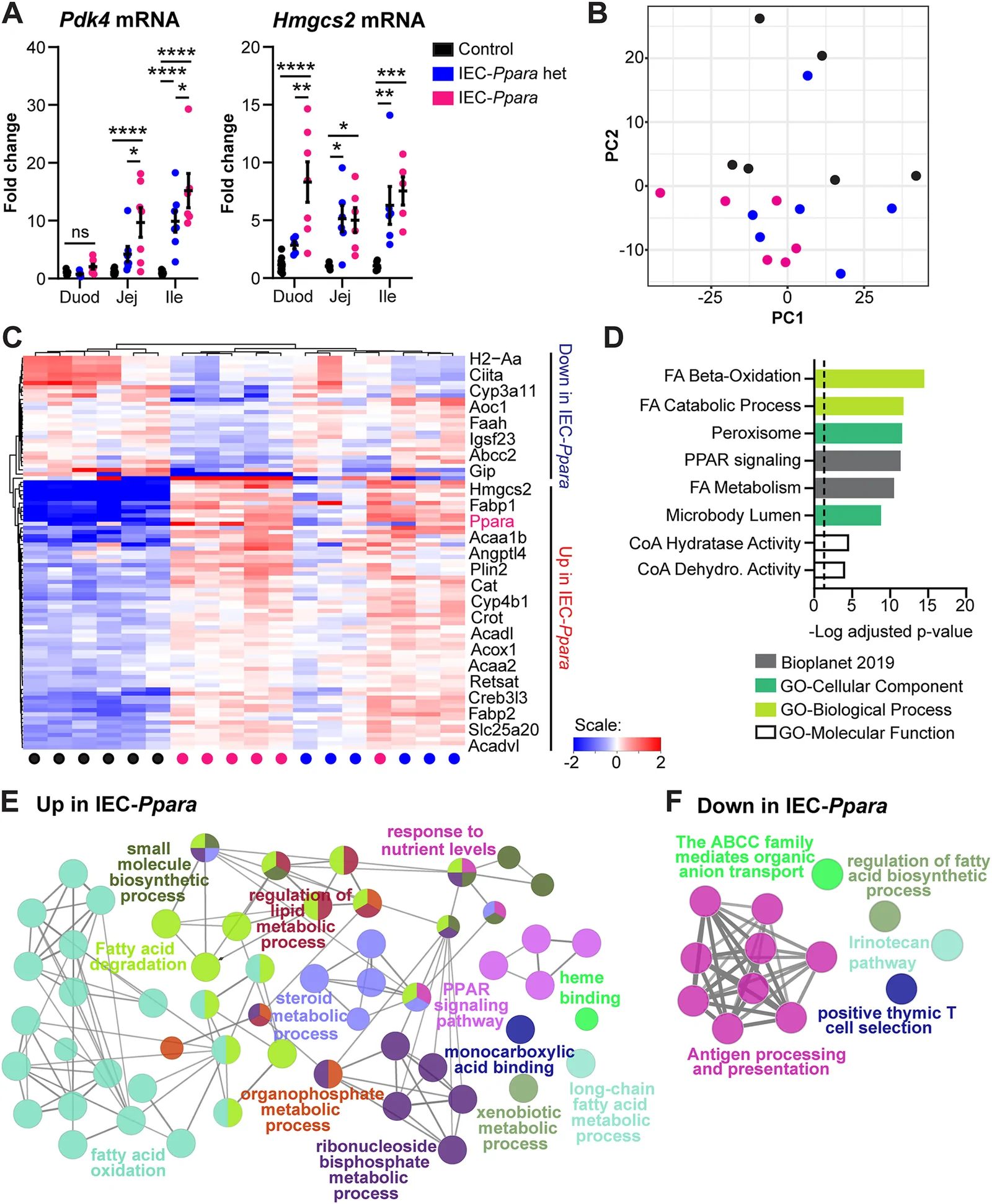
**PPARα signaling activity is enhanced in the ileum of IEC-*Ppara* mice.** (**A**) Gene expression of *Pdk4* and *Hmgcs2* as determined by qRT-PCR in duodenum (Duod), jejunum (Jej), and ileum (Ile) from control (black, n = 11–12), IEC*-Ppara* heterozygous (blue, n = 5–7), and IEC*-Ppara* homozygous (pink, n = 6–7) mice. ∗*P* < 0.05, ∗∗*P* < 0.01, ∗∗∗*P* < 0.001, and ∗∗∗∗*P* < 0.0001, ns (not significant) as determined by 2-way ANOVA and Tukey's multiple comparisons. (**B–F**) RNA-sequencing was performed using whole thickness ileal tissue samples from control (black; n = 6), IEC*-Ppara* heterozygous (blue, n = 6), and IEC*-Ppara* homozygous (pink, n = 6) mice. (**B**) Principal components (PC) analysis. (**C**) Heatmap of normalized gene expression levels for the 95 differentially expressed genes grouped by unsupervised hierarchical clustering, with rows representing genes (selected gene names provided on the right) and columns representing samples. The colored dot below each column indicates sample genotype. Values greater than 0 are relatively upregulated (red) and values less than 0 are relatively downregulated (blue) in IEC-*Ppara* versus control. (**D**) Graph of the top 2 most significantly enriched pathways, GO cellular component, GO biological process, and GO molecular function terms. Dashed line, -log (adjusted *P* = 0.05). (**E-F**) Gene ontology enrichment analysis showing significantly enriched terms from the (**E**) upregulated gene set and (**F**) downregulated gene set in IEC-*Ppara* homozygous vs. control.

We next investigated if the enhanced *Ppara* expression resulted in increased PPARα signaling activity in IEC-*Ppara* intestine. First, we assessed the expression of two known PPARα target genes, *Pdk4* and *Hmgcs2* [14,17]. Both target genes were upregulated across the small intestine of IEC-*Ppara* mice, with the most consistent, allele dosage-dependent upregulation of these two target genes observed in the ileum (Fig. 2A). We therefore next performed RNA-sequencing of ileal tissue to more broadly interrogate the transcriptional programs elicited by enhanced PPARα expression in the intestinal epithelium. Principal component analysis (PCA) showed separation of the control and IEC-*Ppara* samples, with greater separation observed between the controls and the homozygous IEC-*Ppara* samples relative to the heterozygous IEC-*Ppara* samples (Fig. 2B). We identified 95 protein-coding genes that were differentially expressed in at least one pairwise comparison between control, IEC-*Ppara* heterozygous, and IEC-homozygous samples (Table S3). Unsupervised hierarchical clustering of this gene set showed a clear separation of the control and IEC-*Ppara* samples (Fig. 2C). Most differentially expressed genes (67 genes) were upregulated in the IEC-*Ppara* groups and importantly included *Ppara* and several of its known target genes (e.g. *Hmgcs2*, *Fabp1,* and *Pdk4*). The upregulated gene set was significantly enriched for PPAR signaling and fatty acid metabolism pathways (Fig. 2D) as well as for gene ontologies related to fatty acid β-oxidation, response to nutrient levels, steroid metabolic process, organophosphate metabolic process, and xenobiotic metabolic process (Fig. 2D,E). We also observed a smaller set of differentially expressed genes that were downregulated in IEC-*Ppara* ileum (28 genes) (Fig. 2C). The downregulated gene set was significantly enriched for gene ontologies related to antigen processing and presentation (Fig. 2F). The intestinal transcriptional programs elicited by genetic overexpression of *Ppara* were consistent with those previously reported in mice administered WY-14643 [14]. Thus, we concluded that IEC-*Ppara* mice show activation of PPARα signaling activity in the small intestine, even when fed a normal chow diet and without the extraneous delivery of any additional PPARα agonist. Because the heterozygous IEC-*Ppara* transcriptional profiles were heterogeneous, with some samples resembling controls (Fig. 2C), subsequent experiments used only homozygous IEC-*Ppara* mice.

### 3.3 IEC-*Ppara* mice show phenotypes consistent with activated intestinal PPARα signaling

**Fig. 3:**
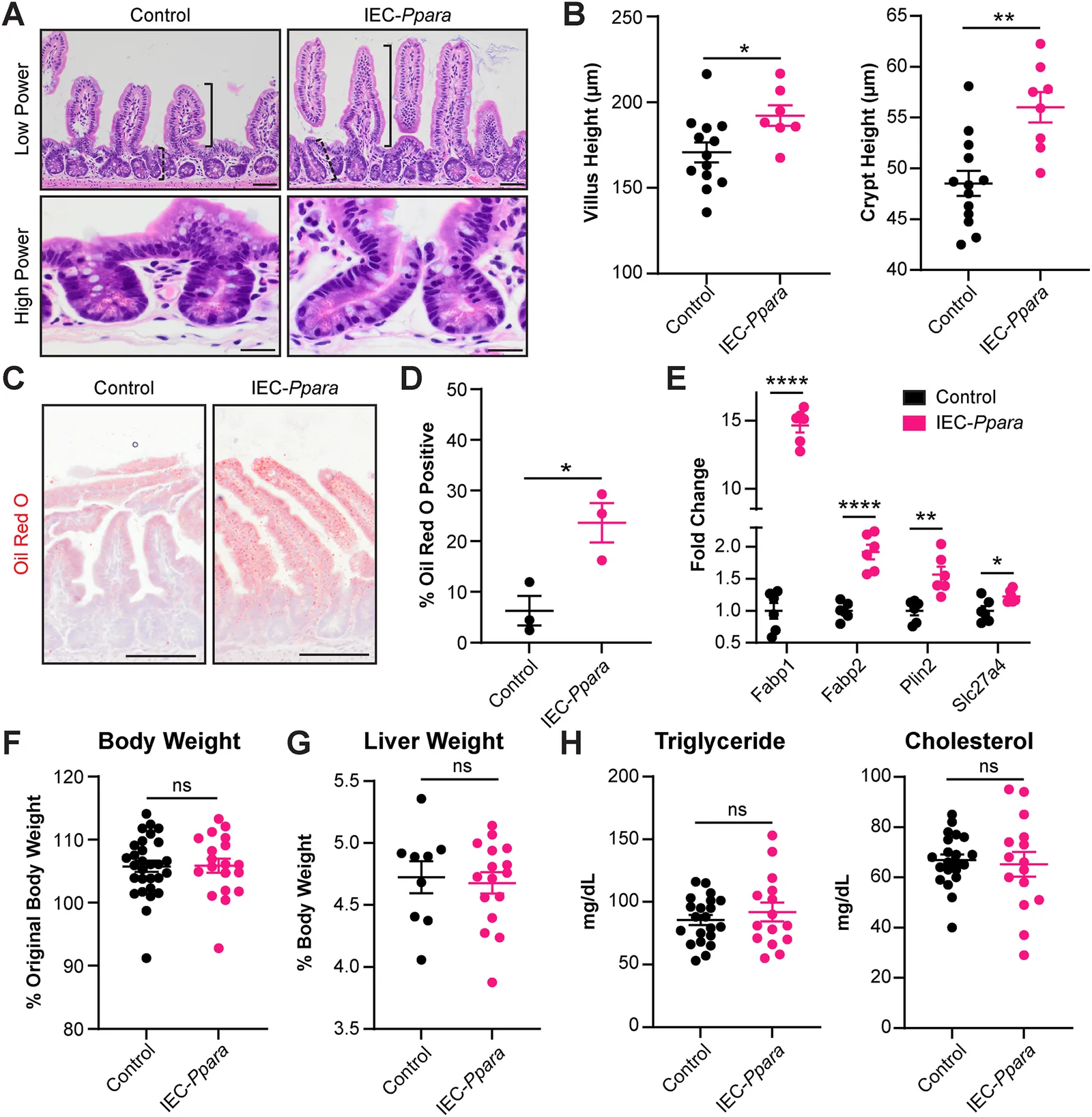
**IEC-*Ppara* mice recapitulate key intestinal phenotypes attributed to PPARα signaling activation.** (**A**) Representative low-power and high-power microscopy images of H&E-stained ileal tissue sections. Solid brackets indicate villus height. Dashed brackets indicate crypt height. (**B**) Quantification of villus height and crypt height from H&E-stained images of control (n = 13) and IEC-*Ppara* (n = 7–8) ileal samples. (**C, D**) Mice were fasted overnight and then administered a bolus of olive oil by oral gavage. Intestinal tissues were collected 2 h post-gavage. (**C**) Representative microscopy images of ileal tissue sections stained with Oil Red O. (**D**) Quantification of Oil Red O-positive area relative to the epithelial area (n = 3). (**E**) Expression of indicated genes in the ileum as determined by RNA-seq (n = 6) and expressed as fold change relative to the controls. (**F**) Quantification of body weight of control (n = 29) and IEC-*Ppara* (n = 19) mice relative to their original body weight. (**G**) Liver weight in control (n = 9) and IEC-*Ppara* (n = 16) mice expressed as percent body weight. (**H**) Quantification of serum triglyceride and cholesterol in control (n = 21) and IEC-*Ppara* (n = 15) mice. For B, D, E− H, ∗*P* < 0.05, ∗∗*P* < 0.01, ∗∗∗∗*P* < 0.0001 or ns, not significant as determined by unpaired t-test.

We next tested if phenotypes previously ascribed to intestinal PPARα signaling, based on studies performed with WY-14643 or *Ppara* loss-of-function mice [14,16], were recapitulated in IEC-*Ppara* mice. As expected, we observed morphometric alterations in IEC-*Ppara* vs. control intestinal tissues, with increased villus height and increased crypt height (Fig. 3A, B). We also observed that IEC-*Ppara* ileal enterocytes had increased capacity to absorb lipids (Fig. 3C, D), in line with the enhanced expression of genes related to lipid uptake and trafficking, such as *Fabp1, Fabp2, Plin2,* and *Slc27a4*, from the RNA-seq analysis (Fig. 3E). However, IEC-*Ppara* mice did not show increased weight gain when compared to controls (Fig. 3F) in male or female mice (Fig. S3A and C). Many of the major metabolic effects of WY-14643 have been attributed to PPARα signaling in hepatocytes, including hepatomegaly and lowered serum triglycerides, and occur within 2-weeks of exposure to WY-14643 [7]. We expected to not observe these effects in IEC-*Ppara* mice. Accordingly, IEC-*Ppara* mice showed no overt changes in liver weight, liver tissue morphology, or hepatocyte lipid storage (Fig. 3G, Fig. S2A–D). Serum triglyceride and cholesterol levels were also not changed in IEC-*Ppara* compared to controls (Fig. 3H), although lowered serum cholesterol was specifically observed in IEC-*Ppara* female mice (Fig. S3B and D). Together, these data demonstrate that IEC-*Ppara* mice recapitulate expected intestinal phenotypes without an overt effect on the liver within the 2-week post-recombination period.

### 3.4 Activated PPARα signaling diminishes GIP-positive enteroendocrine cells

**Fig. 4:**
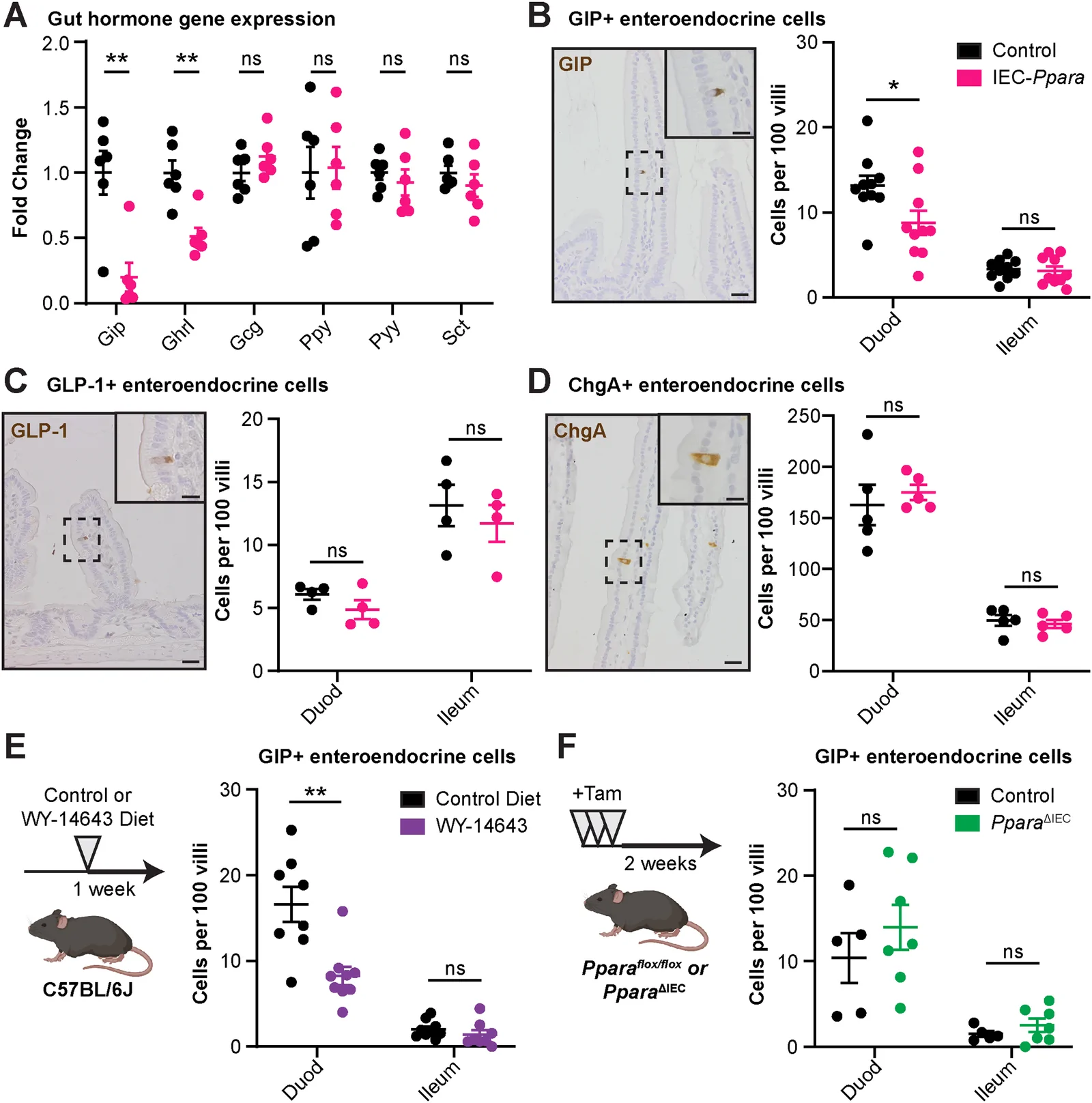
**IEC-*Ppara* mice show reduced GIP-positive but not GLP-1-positive or overall enteroendocrine cell numbers.** (**A**) Expression of indicated genes in the ileum as determined by RNA-seq (n = 6 per genotype) and expressed as fold change relative to the controls. (**B**) Representative image of a GIP+ cell in duodenal tissue at low and high (inset) magnification and quantification of GIP+ cells in duodenum (duod) and ileum (n = 10). (**C**) Representative image of a GLP-1+ cell in ileal tissue at low and high (inset) magnification and quantification of GLP-1+ cells in duodenum (duod) and ileum (n = 4). (**D**) Representative image of a ChgA + cell in duodenal tissue at low and high (inset) magnification and quantification of ChgA + cells (n = 5). (**E**) Mice were fed control diet or control diet containing 0.1% WY-14643 for 1 week followed by quantification of GIP+ cells (n = 8–9). (**F**) *Ppara*^*ΔIEC*^ and control mice were injected with tamoxifen daily for three days, with quantification of GIP+ cells two weeks post-tamoxifen (n = 5–7). For A-F, ∗*P* < 0.05, ∗∗*P* < 0.01, or ns, not significant as determined by unpaired t-test. For B-D, scale bars, 20 μm (low magnification), 10 μm (high magnification).

Unexpectedly, one of the most downregulated genes in IEC-*Ppara* ileum was glucose-dependent insulinotropic polypeptide (*Gip*) (Fig. 2C, Fig. 4A). GIP, along with GLP-1, is a gut incretin hormone with increasingly recognized physiological roles across many tissues [45]. We examined the gene expression of several gut hormones in our RNA-seq data and found that ghrelin (*Ghrl*) and *Tph1* (which encodes the rate-limiting enzyme for serotonin biosynthesis) were also downregulated in IEC-*Ppara* ileal tissue, but *Gcg* (which encodes the precursor for GLP-1), *Ppy*, *Pyy*, and *Sct* were not changed (Fig. 4A). We next determined if the number of GIP-expressing (GIP+) cells was altered in IEC-*Ppara* intestine by immunostaining (Fig. 4B). GIP+ cells (also referred to as K cells) are predominant in the duodenum and rarely present in the ileum [45]. We found that GIP+ cells were significantly reduced in the duodenum but were unchanged in the ileum of IEC-*Ppara* vs. controls (Fig. 4B). In contrast, GLP-1+ cells (also referred to as L cells) are predominant in the ileum and colon and rarely present in the duodenum [46]. GLP-1+ cells were unchanged in IEC-*Ppara* vs. controls (Fig. 4C). Additionally, analysis of the pan-endocrine cell marker Chromogranin A (Chga) supported no change in the overall number of enteroendocrine cells (Fig. 4D). We next aimed to determine if the effect on GIP+ cells was specific to IEC-*Ppara* mice or also occurred in other mouse models with altered PPARα signaling. Because the overexpression of PPARα in IEC-*Ppara* mice could lead to non-physiological effects, we used administration of WY-14643 to wildtype mice through the diet as an alternative approach to activate PPARα signaling. GIP+ cells were also reduced in the duodenum upon exposure to WY-14643 (Fig. 4E). We also tested if *Ppara* loss-of-function in the intestinal epithelium affected GIP+ cells using mice with floxed *Ppara* crossed to *Villin-CreERT2* (herein referred to as *Ppara*^*ΔIEC*^). GIP+ cell number was similar between *Ppara*^*ΔIEC*^ and controls (Fig. 4F). Overall, these data reveal PPARα signaling as a novel regulator of GIP+ enteroendocrine cells.

### 3.5 Reduced GIP hormone but normal glucose tolerance in IEC-*Ppara* mice

**Fig. 5:**
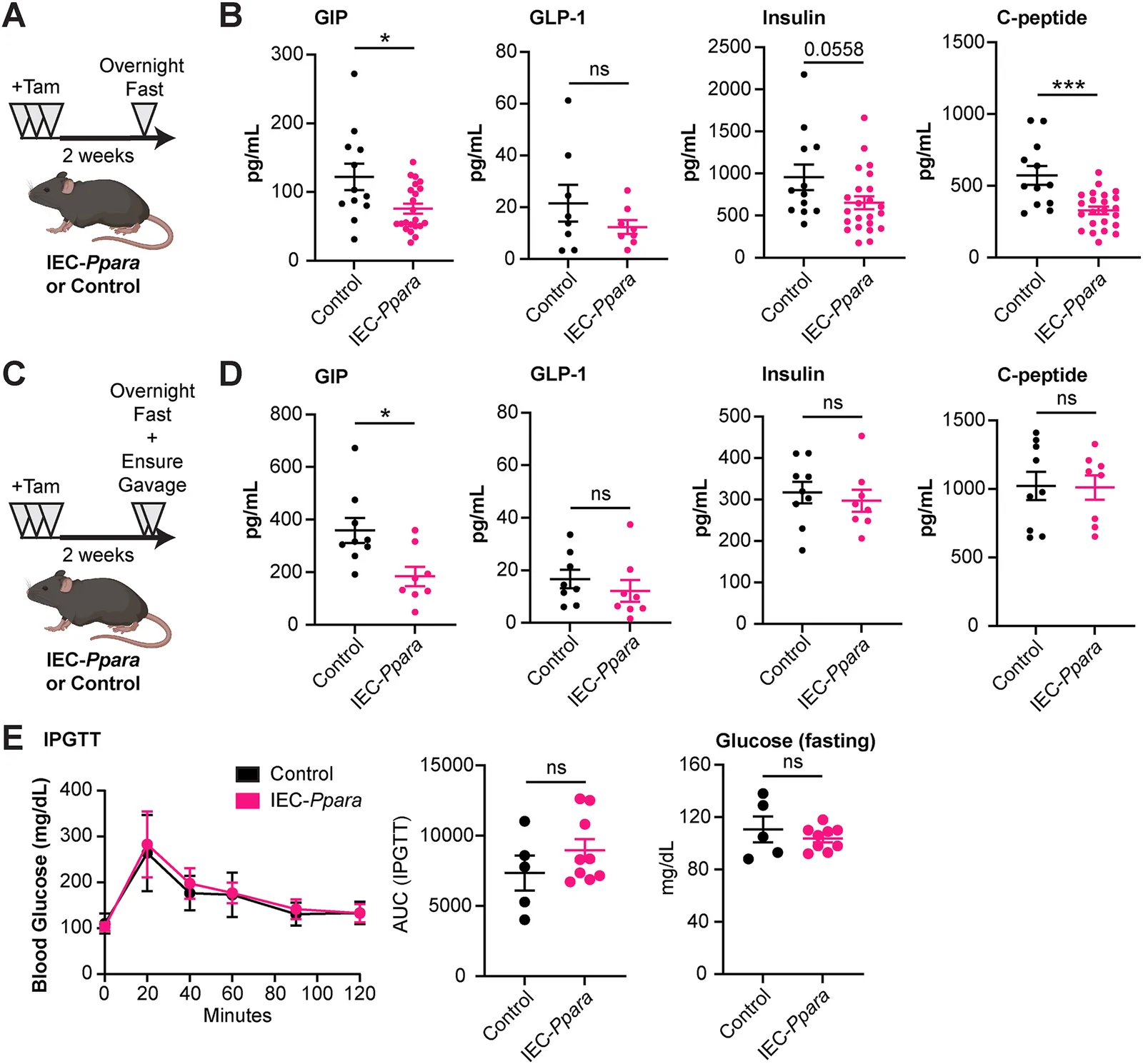
**Diminished GIP hormone, but not GLP-1 hormone, in IEC*-Ppara* mice.** (**A**) Schematic of experiment design to collect serum analyzed in (B). (**B**) Quantification of fasting GIP, GLP-1, insulin, and C-peptide (n = 8–23). (**C**) Schematic of experiment design to collect serum analyzed in (D). Serum was collected 30 min after Ensure administration. (**D**) Quantification of post-feeding GIP, GLP-1, insulin, and C-peptide (n = 8–9). (E) Intraperitoneal glucose tolerance test (IPGTT; left), the associated area under the curve (AUC; middle), and fasting blood glucose levels (right) (n = 5–9). ∗*P* < 0.05, ∗∗∗*P* < 0.001, or ns, not significant as determined by unpaired t-test.

Given that IEC-*Ppara* mice had reduced GIP+ cells, we next asked if this was sufficient to impact GIP hormone levels. For this, we assessed hormone levels in serum collected from mice that were fasted overnight with or without oral administration of Ensure nutrition shake in the morning to stimulate hormone secretion (Fig. 5A,C). In IEC-*Ppara* relative to controls, GIP hormone levels were significantly reduced in both the fasted and refed states (Fig. 5B,D). Circulating GLP-1 hormone levels were unchanged (Fig. 5B,D). Other hormones produced by the gut (Ghrelin, PYY), pancreas (amylin, PP), or adipose tissue (resistin, leptin) with roles in glucose homeostasis and appetite regulation were also unchanged in IEC-*Ppara* vs. controls in either the fasted or refed state (Fig. S4A–S4D).

Because of GIP's known role as an incretin, we next investigated glucose metabolism in IEC-*Ppara* and control mice. We observed a trend towards reduced insulin and significantly reduced C-peptide in fasted IEC-*Ppara* vs. control serum, but no difference in insulin or C-peptide after oral administration of Ensure nutritional shake (Fig. 5B,D). Moreover, IEC-*Ppara* mice showed a similar glucose excursion response to an intraperitoneal glucose tolerance test as well as similar fasting blood glucose levels to controls (Fig. 5E), supporting functional maintenance of pancreatic β-cell insulin secretion.

### 3.6 Intestinal PPARα signaling increases food intake

**Fig. 6:**
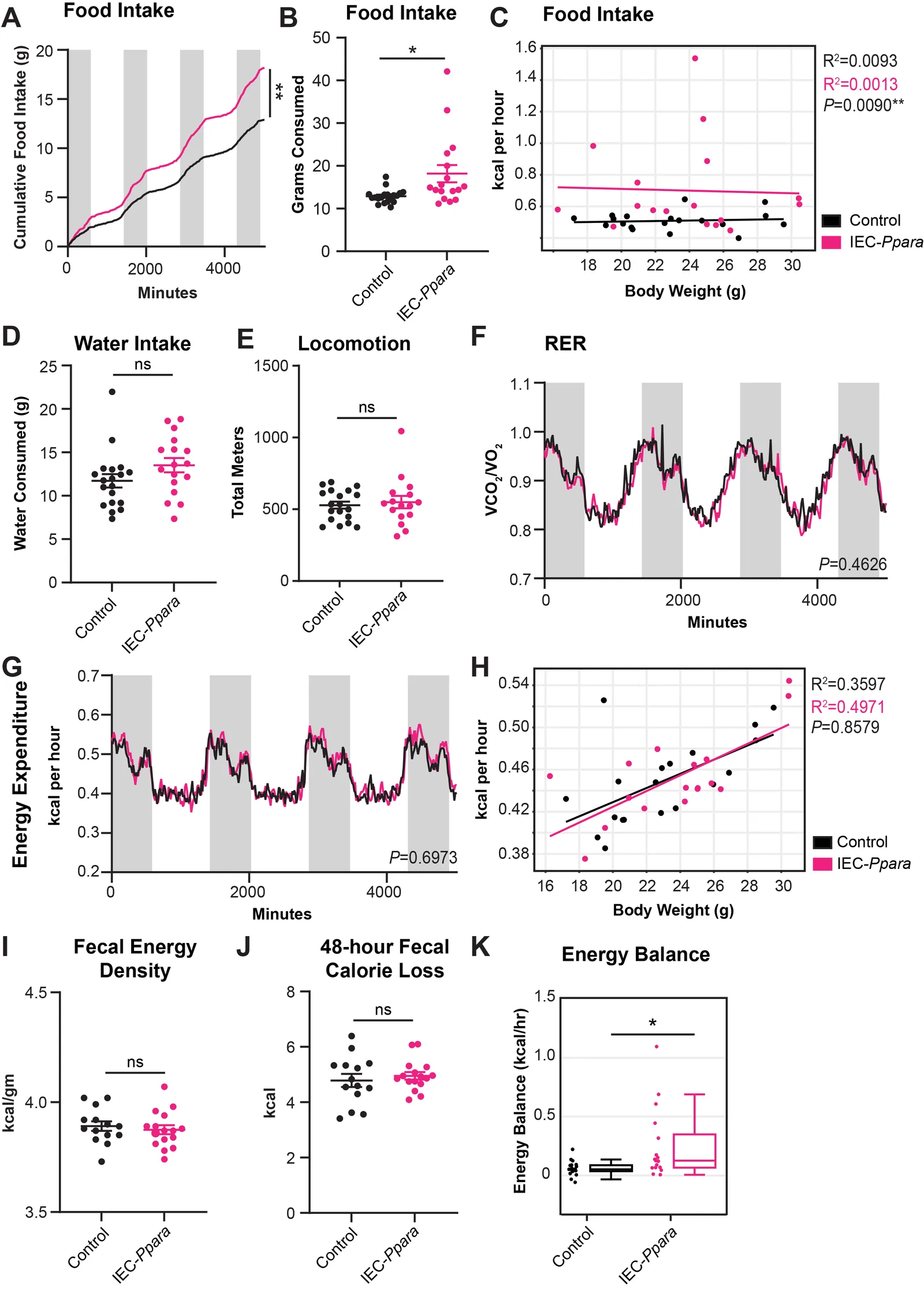
**IEC-*Ppara* mice have increased food intake and positive energy balance.** (**A-H, K**) Mice were singly housed in metabolic cages and analyzed over an 83.5-hour period (n = 16–19). Cumulative food intake graphed over time (**A**), in individual mice (**B**), and as X–Y plot of analysis of covariance (ANCOVA) using body weight as a covariate (**C**). (**D**) Cumulative water intake. (**E**) Cumulative directed locomotion. (**F**) Respiratory exchange ratio (RER) over time. Energy expenditure over time (**G**) and as X–Y plot of ANCOVA using body weight as a covariate (**H**). (**I, J**) Bomb calorimetry of fecal pellets collected over 48-hour period (n = 14–16). (**I**) Energy density of fecal pellets. (**J**) Total fecal calorie loss over the 48-hour period. (**K**) Energy balance determined by CalR. For A, F, and G, ∗∗*P* < 0.01, or exact p-value as determined by 2-way repeated measures ANOVA. Dark photoperiod is represented by gray rectangles. For B, D, E, I, and J, ∗*P* < 0.05 or ns, not significant as determined by t-test. For C and H, ∗∗*P* < 0.01 or the exact *P*-value determined by ANCOVA are provided. R^2^ values are shown for control (black text) or IEC-*Ppara* (pink text). For K, ∗*P* < 0.05 as determined by ANOVA in CalR.

It was recently reported that GIP secretion diminishes food intake [47]. Thus, coincident with reduced GIP hormone secretion, we surmised that food intake may be increased in IEC-*Ppara* vs. control mice. To test this, we singly housed mice in metabolic cages. We indeed observed significantly increased food intake in IEC-*Ppara* mice (Fig. 6A,B), and this effect was independent of body weight (Fig. 6C). When we broke this phenotype down by sex, we found that the effect was significant in female mice and trended upwards in male mice (Fig. S5A–D). Water intake was similar between the genotype groups (Fig. 6D, Fig. S5E). Because we observed increased food intake but no change in body weight (Fig. 3F), we expected that we might observe a change in activity or metabolic rate. However, indirect calorimetry experiments did not show any differences in the directed locomotion, respiratory exchange ratio, oxygen consumption, carbon dioxide production, or energy expenditure between IEC-*Ppara* and control mice of either sex at this time point (Fig. 6E–H, Fig. S5F–J, Fig. S6A–D). We assessed the tissue morphology and weights of several fat pads and, consistent with the indirect calorimetry results, observed no overt differences between IEC-*Ppara* and control mice (Fig. S7). We also performed bomb calorimetry of fecal pellets and found no differences in fecal energy density or calorie loss (Fig. 6I,J), indicating that mice are similarly absorbing calories. Together, these data support a significant increase in the energy balance of IEC-*Ppara* mice at this experimental time (Fig. 6K). Overall, our findings support diminished GIP+ enteroendocrine cells, diminished GIP hormone secretion, and increased food intake as early events downstream of PPARα signaling in the intestinal epithelium.

## 4 Discussion

This is the first report describing a new experimental animal model that enables spatial and temporal control of *Ppara* overexpression, the CAG-*Ppara*,-*EGFP* mouse. CAG-*Ppara*,-*EGFP* mice represent an important addition to the existing research toolbox available for studying PPARα biology. This targeted transgenic mouse strain can be crossed to any available Cre driver, providing investigators with a unique opportunity to interrogate tissue-specific effects of PPARα activation while avoiding potentially confounding systemic or developmental effects. Using *Villin-CreERT2* to direct *Ppara* overexpression to the adult intestinal epithelium, we observed up to ∼4-fold increase in *Ppara* mRNA expression in the small intestinal tissue. This increase is similar to that observed with obesity in human intestinal biopsy tissues and after long-term high fat diet in mouse intestinal tissues [17]. Thus, the induction levels achieved with this mouse strain are reflective of the physiological range of *Ppara* mRNA. Despite the achievement of a relatively modest overexpression of PPARα with this novel mouse strain, it is important to acknowledge that overexpression models can produce non-physiological effects. Thus, we envision that this research tool would be applied in concert with alternative models, including PPARα agonism, antagonism, genetic loss-of-function, and other tunable expression (e.g. degradation tag, CRISPRa, CRISPRi) approaches to dissect tissue-specific PPARα signaling responses.

Our findings show that the intestinal epithelium is highly sensitive to relatively modest increases in PPARα receptor abundance. We observed a robust transcriptional response in the IEC-*Ppara* intestine characterized by upregulation of known PPARα target genes and enrichment for pathways associated with lipid metabolism and a shifted metabolic state. Among the subset of downregulated genes, we observed enrichment for pathways related to antigen presentation. The concordance of these transcriptional profiles with those previously reported for mice treated with WY-14643 confirms that the IEC-*Ppara* model recapitulates canonical PPARα signaling responses without additional administration of a systemic agonist or ligand [14]. We surmise that dietary or intracellular lipid sources are the most likely ligand source in this model, and future studies should test whether distinct effects occur if these mice are exposed to alternative ligand sources via diet or via agonist administration. Although we did not observe gene expression differences in related family members *Ppard* or *Pparg*, we cannot rule out that the functional activity of these ligand-induced transcription factors might be altered in IEC-*Ppara* intestine [48]. Because there are many shared gene targets of the PPARs, it is difficult to determine this from transcriptional analysis alone. Future studies applying chromatin, epigenomic, or *in vitro* cell culture approaches would clarify whether the observed transcriptional changes in IEC-*Ppara* intestinal tissue reflect direct binding of PPARα to DNA, RXR heterodimer competition, or secondary responses to metabolic remodeling or epithelial-immune crosstalk.

Consistent with previous reports using WY-14643 treatment or *Ppara*^ΔIEC^ mice [14,16], we observed increased villus height, increased crypt depth, and enhanced enterocyte lipid uptake following an oral lipid challenge in IEC-*Ppara* mice. These data provide further evidence that PPARα signaling in intestinal epithelial cells directly regulates intestinal surface area and the lipid transport capacity of enterocytes. Prior studies have argued that the volume of food intake, independent of total caloric intake, is a critical regulator of organ size [16,19,22,49,50]. As a supporting example, the *db/db* and *ob/ob* mouse models of obesity are hyperphagic and show increased intestinal weight, length, and epithelial surface area on standard rodent chow [16,19]. However, *Ppara*^ΔIEC^ mice had similar food intake and decreased villus height vs. controls [16], WY-14643-treated mice had diminished food intake and increased villus height vs. controls [7], and IEC-*Ppara* mice had increased food intake and increased villus height vs. controls. Thus, PPARα signaling activation appears to be dominant over food intake in the expansion of intestinal surface area, potentially through the actions of PPARα on intestinal stem cell activity [18].

A key finding of this study was the identification of intestinal PPARα signaling as a potential regulator of the incretin hormone GIP. *Gip* was one of the most down-regulated genes in IEC-*Ppara* small intestine. We observed a specific reduction in the number of GIP+ enteroendocrine cells in two independent mouse models of activated PPARα signaling, IEC-*Ppara* and WY-14643-treated mice. We did not observe a significant difference in GIP+ cells in *Ppara*^ΔIEC^ intestine; however, there did appear to be a trend towards increased GIP+ cells that warrants additional investigation, particularly whether there might be compensation from PPARγ or PPARδ in the loss-of-function context. Future experiments in which WY-14643 is administered to *Ppara*^ΔIEC^ and littermate control mice are needed to further test whether PPARα signaling in the intestinal epithelium is required for these observed effects on GIP-expressing cells and to rule out potential off-target effects of WY-14643. Whereas PPARδ activation has been reported to enhance GLP-1 transcription and hormone release by enteroendocrine cells [51], to our knowledge, PPARα has not been previously linked to the regulation of enteroendocrine cells. Interestingly, we did not observe a change in GLP-1-expressing enteroendocrine cells or the overall number of enteroendocrine cells in IEC-*Ppara* small intestine, suggesting that other endocrine cell subtypes may be increased. There are several plausible mechanisms by which intestinal PPARα signaling could regulate GIP+ cells or GIP secretion. PPARα might directly repress GIP transcription in K cells; we have noted putative PPAR response elements in the genomic sequence near the GIP gene. Alternatively, PPARα might shift differentiation of enteroendocrine progenitors away from K cells through its influence on intestinal stem and progenitor cell activity [18] or on signaling pathways (e.g. mTOR, Notch, Wnt) that regulate enteroendocrine cell differentiation [52]. Altered basolateral release of lipids, chylomicrons, or bile acids by the intestinal epithelium can affect enteroendocrine hormone secretion [53,54]. Finally, PPARα signaling modulation of the gut microbiome [55] could indirectly affect GIP expression or secretion [56]. Future studies will determine which of these mechanisms is most likely.

GIP, along with GLP-1, is an incretin hormone that stimulates insulin release from pancreatic β-cells, in addition to other roles in energy homeostasis [45,57]. GIP and GLP-1 synthesis and secretion is stimulated by nutrients in the gut lumen, including glucose, amino acids, fatty acids, and bile acids [57]. We found that circulating levels of GIP, but not GLP-1, were reduced in the serum of IEC-*Ppara* mice in the fasted state and after stimulation of hormone secretion via a mixed meal. The reduction in GIP hormone is congruent with reduced GIP+ enteroendocrine cells in IEC-*Ppara* mice. However, it is also possible that PPARα signaling regulates GIP hormone secretion from K cells, which we did not test. An additional limitation of our study is that we did not determine GIP hormone levels in WY-14643-treated mice or *Ppara*^ΔIEC^ mice. In fasted IEC-*Ppara* mice, we observed a trend towards reduced insulin and a significant reduction in C-peptide, a more stable indicator of insulin secretion. Yet IEC-*Ppara* mice showed normal fasting glucose levels and glucose excursion during an IPGTT. These results are aligned with previous reports. For example, in a mouse model with diphtheria toxin-mediated ablation of GIP-expressing cells, glucose tolerance was similar relative to controls in an IPGTT but impaired in an OGTT [58]. Furthermore, single incretin receptor knockout mice (i.e. either GIPR or GLP-1R) generally show only modest effects in GTTs; loss of both GIPR and GLP-1R signaling was required to impair glycemic excursion [59,60]. GIPR knockout mice also show upregulation of compensatory mechanisms that render pancreatic islet β-cells more sensitive to GLP-1 hormone [61]. Thus, we surmise that the unchanged GLP-1-positive cells and hormone levels may be sufficient to compensate for the reduced GIP levels in IEC-*Ppara* mice, although this warrants a deeper exploration.

A recent study utilizing the DREADD system to stimulate hormone release from GIP-expressing K cells beautifully demonstrated that intestinal GIP secretion suppresses food intake [47]. Thus, our finding that IEC-*Ppara* mice consumed more food is in alignment with the lower serum GIP hormone levels observed in this model. As WY-14643-treated mice also showed lower GIP+ cells but have reduced food intake, it appears that the hepatocyte-mediated effect of WY-14643 outweighs the effect of GIP on satiety [7]. This finding reinforces the point that the CAG-*Ppara*,-*EGFP* mouse model can enable discovery of tissue-specific phenotypes that might otherwise be masked by the systemic metabolic remodeling induced by WY-14643. Despite increased food volume and caloric intake, IEC-*Ppara* mice maintained normal body weight. We assumed that this would be accomplished by a shift in energy expenditure, respiratory exchange ratio, locomotion, or fecal caloric density, but we did not observe shifts in these parameters and instead found a heightened energy balance in the IEC-*Ppara* mice. Accordingly, we also did not observe any overt changes in liver or fat pad weights or histology. A limitation of our study is that we only assessed these metabolic parameters at 2 weeks post-tamoxifen treatment. A longer-term study might reveal adaptations to maintain systemic energy balance in IEC-*Ppara* mice. Together, our data supports a model in which diminished GIP+ enteroendocrine cells is an early event downstream of intestinal PPARα signaling leading to diminished GIP hormone levels and increased food intake.

To combat rising prevalences of metabolic dysfunction, obesity, and related co-morbidities, new therapeutic strategies are rapidly emerging. In addition to GLP1R-GIPR agonists, PPAR agonists are also under active investigation as therapies for metabolic diseases [[62], [63], [64]]. Notably, a recent preclinical study of a unimolecular quintuple agonist for GLP-1/GIP/PPARα/PPARδ/PPARγ enhanced metabolic benefits at lower drug doses by targeting the drug to GLP1R/GIPR-expressing cells [12]. The quintuple agonist outperformed GLP1R/GIPR co-agonism in reducing body weight, food intake, and hyperglycemia in obese mouse models. Despite these advances, the tissue-specific mechanisms by which PPAR agonists regulate glucose metabolism and systemic energy balance remain poorly defined. Our study of IEC-*Ppara* mice suggests that intestinal PPARα activation suppresses the gut hormone GIP, thereby altering systemic energy balance. This represents a new, tissue-specific mechanism by which PPARα functions as a metabolic regulator via a gut endocrine hormone axis. Future studies can explore whether this PPARα-GIP signaling axis contributes to the inter-individual variation observed in glucose homeostasis and GLP1R-GIPR agonist therapy responses in patients.

## CRediT authorship contribution statement

**Jacob J. Socha:** Writing – review & editing, Writing – original draft, Visualization, Methodology, Investigation, Formal analysis, Conceptualization. **Swathi Pavithran:** Writing – review & editing, Investigation. **Courtney A. Burger:** Writing – review & editing, Resources, Methodology. **Rebekah Karns:** Writing – review & editing, Formal analysis. **Avelina W. Lee:** Writing – review & editing, Investigation, Formal analysis. **Richard Lang:** Writing – review & editing, Resources, Funding acquisition. **Maria E. Moreno-Fernandez:** Writing – review & editing, Methodology, Investigation. **Stephen W. Standage:** Writing – review & editing, Resources, Methodology, Funding acquisition. **Kelli L. VanDussen:** Writing – review & editing, Writing – original draft, Visualization, Supervision, Methodology, Funding acquisition, Formal analysis, Conceptualization.

## Ethics declaration

This study was conducted in accordance with the ARRIVE (Animal Research: Reporting of In Vivo Experiments) guidelines. This study was approved by the Cincinnati Children's Hospital Medical Center Institutional Animal Care and Use Committee.

## Funding sources

The VanDussen lab acknowledges support from the National Institutes of Health (R01 DK132043) and The Leona M. and Harry B. Helmsley Charitable Trust to Cincinnati Children's Hospital Medical Center. This work was also supported in part by NIH P30 DK078392 Integrative Morphology Core of the Digestive Diseases Research Core Center in Cincinnati. The Genome Technology Access Center at the McDonnell Genome Institute at Washington University School of Medicine is partially supported by NIH P30 CA91842. The Standage lab acknowledges support from NIH K08 HL133377. The Lang lab acknowledges support from NIH, from the National Eye Institute (grants EY032029, EY032752, EY032566, EY034456), from the National Institute of General Medical Sciences (grant GM152641) and from the Emma and Irving Goldman Scholar Endowed Chair and from the Cincinnati Children's Hospital Research Foundation Academic and Research Committee.

## Declaration of competing interest

The authors declare the following financial interests/personal relationships which may be considered as potential competing interests: Kelli VanDussen reports financial support was provided by National Institutes of Health. Stephen W. Standage reports financial support was provided by National Institutes of Health. Richard Lang reports financial support was provided by National Institutes of Health. Kelli L VanDussen reports financial support was provided by The Leona M and Harry B Helmsley Charitable Trust. If there are other authors, they declare that they have no known competing financial interests or personal relationships that could have appeared to influence the work reported in this paper.

## Data Availability

Data will be made available on request.
