# Supplementary material for: Conditional overexpression of PPARα in intestinal epithelium diminishes GIP enteroendocrine cells and circulating hormone levels
Source: Mol Metab. 2026 Aug 13;112:102429. doi: 10.1016/j.molmet.2026.102429 (PMC13542957; doi:10.1016/j.molmet.2026.102429)
Supplement: Supplementary file 1 [file mmc1.pdf]

Supplementary Table S1. Oligonucleotide sequences used for PCR genotyping.

Primer	Sequence
PparaTGLHA	CTTGGGACTCTTCCTGGC
PparaTGRHA	GGTACCTATGGAGACTGTGC
P1 (VS7255)	GGCTTCTGGCGTGTGACCG
P2 (VS4520)	CCTGAACCTGAAACATAAAATGAATGC
P3 (VS7615)	ACTCCATATATGGGCTATGAACTAATGACC
P4 (VS7532)	CAATGCTGAAATGTCCCACCAGCC
P5 (VS7645)	GTTTGCTCCCAAACGGAGTCATTCC
P6 (VS7616)	AGAGAATAGGAACTTCATCGATCTGGC
P7 (VS6743)	CAGCCCTGCACAGAATTGTCAG
P8 (VS7584)	CATATAGACAAACGCACACCGGCC

Supplementary Table S2. Oligonucleotide sequences used for quantitative reverse transcriptase PCR.

Gene	Forward Primer (5' to 3')	Reverse Primer (5' to 3')
Fabp1	ATGAACTTCTCCGGCAAGTACC	CTGACACCCCCTTGATGTCC
Gapdh	TCAAGAAGGTGGTGAAGCAGG	TATTATGGGGGTCTGGGATGG
Hmgcs2	AGTCCAGCTACTGGGATGGT	ACGCGTTCTCCATGTGAGTT
Pdk4	AGGGAGGTCGAGCTGTTCTC	GGAGTGTTCACTAAGCGGTCA
Ppara	GCGTACGGCAATGGCTTTAT	AGCCTATGTTTAGAAGGCCAGG
Ppard	TATGCGCATGGGACTCACTC	TGGCTGTTCCATGACTGACC
Pparg	TTCGCTGATGCACTGCCTAT	GGAATGCGAGTGGTCTTCCA

Supplementary Table S3. List of differentially expressed genes in IEC-*Ppara* vs. control ileum.

EnsID	Symbol	Homo vs. Het unadj. P-value	Homo vs. Het FDR-adj. P-value	Homo vs. Het Fold Change	Het vs. Control unadj. P-value	Het vs. Control FDR-adj. P-value	Het vs. Control Fold Change	Homo vs. Control unadj. P-value	Homo vs. Control FDR-adj. P-value	Homo vs. Control Fold Change
ENSMUSG00000014351	Gip	0.00112	0.77652	-8.447	0.88381	0.99994	-1.055	0.0008	0.13522	-8.913
ENSMUSG00000031972	Acta1	0.38809	0.99994	-1.297	0.01374	0.99994	-2.078	0.00089	0.14831	-2.696
ENSMUSG00000022504	Ciita	0.32983	0.99994	-1.272	0.01859	0.99994	-1.752	0.00091	0.14859	-2.229
ENSMUSG00000058618	Defa39	0.00006	0.30066	-1.757	0.17354	0.99994	-1.208	<0.00001	0.00004	-2.122
ENSMUSG00000079180	Mptx2	0.04509	0.99994	-1.46	0.04581	0.99994	-1.451	0.00006	0.02048	-2.118
ENSMUSG00000037649	H2-DMa	0.31632	0.99994	-1.194	0.00405	0.59116	-1.611	0.00011	0.02974	-1.924
ENSMUSG00000079547	H2-DMb1	0.70482	0.99994	-1.074	0.00165	0.32425	-1.745	0.00043	0.08144	-1.874
ENSMUSG00000028278	Rragd	0.00099	0.73138	-1.65	0.60677	0.99994	-1.073	0.00014	0.03474	-1.771
ENSMUSG00000024610	Cd74	0.35828	0.99994	-1.137	0.00192	0.35962	-1.539	0.00006	0.01956	-1.749
ENSMUSG00000073421	H2-Ab1	0.43491	0.99994	-1.11	0.00146	0.29295	-1.525	0.00007	0.02308	-1.693
ENSMUSG00000036594	H2-Aa	0.40203	0.99994	-1.126	0.00348	0.54179	-1.504	0.00017	0.03841	-1.693
ENSMUSG00000056035	Cyp3a11	0.00182	0.99994	-2.039	0.33238	0.99994	1.239	0.03068	0.99974	-1.646
ENSMUSG00000041301	Cftr	0.00024	0.31138	-1.615	0.99167	0.99994	-1.001	0.00023	0.04749	-1.617
ENSMUSG00000060586	H2-Eb1	0.49902	0.99994	-1.099	0.00659	0.83322	-1.457	0.00069	0.12025	-1.602
ENSMUSG00000074437	Defa29	0.00144	0.94251	-1.455	0.41114	0.99994	-1.1	0.00006	0.02001	-1.601
ENSMUSG00000025194	Abcc2	0.00039	0.38141	-1.658	0.67136	0.99994	1.062	0.00177	0.23257	-1.562
ENSMUSG00000027792	Bche	0.0006	0.53677	-1.434	0.82619	0.99994	1.022	0.00126	0.1842	-1.403
ENSMUSG00000044461	Shisa2	0.00189	0.99994	-1.704	0.15318	0.99994	1.265	0.0891	0.99974	-1.347
ENSMUSG00000022026	Olfm4	0.00163	0.99994	-1.264	0.45364	0.99994	-1.056	0.0001	0.02695	-1.335
ENSMUSG00000021208	Ifi27l2b	0.00021	0.31138	-1.342	0.61857	0.99994	1.04	0.00131	0.18697	-1.291
ENSMUSG00000030934	Oat	0.00064	0.53677	-1.255	0.96176	0.99994	-1.003	0.00053	0.096	-1.259
ENSMUSG00000071203	Naip5	0.0019	0.99994	-1.272	0.77287	0.99994	1.022	0.0048	0.50698	-1.244
ENSMUSG00000034171	Faah	0.00015	0.30066	-1.321	0.35012	0.99994	1.068	0.004	0.44166	-1.237
ENSMUSG00000005373	Mlxipl	0.00018	0.30066	-1.348	0.19579	0.99994	1.107	0.01385	0.88646	-1.218
ENSMUSG00000040498	Igsf23	0.0001	0.30066	-1.424	0.04884	0.99994	1.193	0.05361	0.99974	-1.194
ENSMUSG00000069515	Lyz1	0.00035	0.38141	-1.297	0.05037	0.99994	1.153	0.10587	0.99974	-1.125
ENSMUSG00000004655	Aqp1	0.00098	0.73138	-1.209	0.16734	0.99994	1.081	0.05437	0.99974	-1.118
ENSMUSG00000029811	Aoc1	0.00014	0.30066	-1.232	0.0276	0.99994	1.128	0.10803	0.99974	-1.092
ENSMUSG00000022479	Vdr	0.03388	0.99994	-1.13	0.00087	0.20167	1.211	0.22682	0.99974	1.073
ENSMUSG00000059447	Hadhb	0.28618	0.99994	-1.081	0.00052	0.13884	1.298	0.01588	0.97752	1.2
ENSMUSG00000036138	Acaa1a	0.45327	0.99994	-1.05	0.00022	0.06553	1.275	0.00321	0.37179	1.214
ENSMUSG00000023232	Serinc2	0.36745	0.99994	1.057	0.01409	0.99994	1.169	0.00078	0.13342	1.236
ENSMUSG00000053898	Ech1	0.81104	0.99994	-1.016	0.00059	0.15247	1.274	0.00137	0.1926	1.253
ENSMUSG00000025745	Hadha	0.4523	0.99994	-1.045	<0.00001	0.00187	1.315	0.00013	0.03154	1.258
ENSMUSG00000005514	Por	0.449	0.99994	-1.071	0.00065	0.15679	1.369	0.00793	0.69632	1.277
ENSMUSG00000056429	Tgoln1	0.39949	0.99994	1.057	0.00373	0.56591	1.216	0.00018	0.03954	1.286
ENSMUSG00000027809	Etfdh	0.58741	0.99994	-1.042	0.00014	0.04578	1.348	0.00108	0.16854	1.294
ENSMUSG00000026385	Dbi	0.70774	0.99994	1.026	0.00067	0.15827	1.263	0.00016	0.03651	1.295
ENSMUSG00000020458	Rtn4	0.08744	0.99994	1.125	0.03747	0.99994	1.16	0.00015	0.03528	1.305
ENSMUSG00000036880	Acaa2	0.82784	0.99994	-1.017	0.00054	0.14176	1.33	0.00116	0.17785	1.308
ENSMUSG00000020777	Acox1	0.67455	0.99994	1.032	0.00138	0.28694	1.274	0.00029	0.059	1.314
ENSMUSG00000022853	Ehhadh	0.34652	0.99994	-1.098	0.00033	0.09413	1.443	0.00799	0.69632	1.314
ENSMUSG00000027187	Cat	0.32887	0.99994	-1.075	<0.00001	0.00185	1.417	0.00027	0.05458	1.317
ENSMUSG00000026003	Acadl	0.33121	0.99994	1.077	0.0064	0.83155	1.238	0.00022	0.04637	1.334
ENSMUSG00000028127	Abcd3	0.70197	0.99994	-1.03	0.00002	0.00768	1.401	0.00011	0.0291	1.36
ENSMUSG00000003623	Crot	0.66797	0.99994	1.039	0.00064	0.15639	1.374	0.00012	0.03071	1.427
ENSMUSG00000010025	Aldh3a2	0.95247	0.99994	1.004	<0.00001	0.0014	1.421	<0.00001	0.00074	1.427
ENSMUSG00000027359	Slc27a2	0.39482	0.99994	-1.107	0.00025	0.07225	1.589	0.00468	0.49808	1.436
ENSMUSG00000037419	Endod1	0.00377	0.99994	1.307	0.29335	0.99994	1.105	0.00008	0.02381	1.444
ENSMUSG00000018900	Slc22a5	0.4099	0.99994	1.072	0.00046	0.12603	1.363	0.00002	0.0054	1.461
ENSMUSG00000006800	Sulf2	0.02734	0.99994	1.244	0.10649	0.99994	1.18	0.00013	0.03294	1.468
ENSMUSG00000029153	Ociad2	0.00352	0.99994	1.403	0.59568	0.99994	1.067	0.00056	0.09959	1.497
ENSMUSG00000018574	Acadvl	0.61683	0.99994	1.038	<0.00001	0.00187	1.447	<0.00001	0.00017	1.503
ENSMUSG00000027801	Tm4sf4	0.19377	0.99994	-1.147	<0.00001	0.00051	1.74	0.00025	0.05221	1.518
ENSMUSG00000024507	Hsd17b4	0.50414	0.99994	1.049	<0.00001	0.00003	1.505	<0.00001	<0.00001	1.579
ENSMUSG00000028494	Plin2	0.36094	0.99994	1.088	0.00011	0.0354	1.452	<0.00001	0.00073	1.58
ENSMUSG00000028713	Cyp4b1	0.91135	0.99994	-1.013	0.00005	0.01827	1.63	0.00008	0.02413	1.609
ENSMUSG00000035041	Creb3l3	0.28777	0.99994	-1.15	<0.00001	0.00185	1.85	0.00037	0.07247	1.609
ENSMUSG00000027698	Nceh1	0.42988	0.99994	1.105	0.00289	0.49864	1.513	0.00017	0.03841	1.672
ENSMUSG00000025277	Abhd6	0.25034	0.99994	1.104	<0.00001	0.00187	1.517	<0.00001	0.00001	1.675
ENSMUSG00000037440	Vnn1	0.29494	0.99994	1.096	<0.00001	0.00051	1.562	<0.00001	<0.00001	1.712
ENSMUSG00000032602	Slc25a20	0.11	0.99994	1.12	<0.00001	0.00001	1.546	<0.00001	<0.00001	1.731
ENSMUSG00000006342	Susd2	0.35404	0.99994	1.124	0.00063	0.15639	1.553	0.00001	0.00514	1.745
ENSMUSG00000028383	Hsdl2	0.53828	0.99994	1.066	0.00001	0.00438	1.661	<0.00001	0.00027	1.772
ENSMUSG00000032883	Acsl3	0.88598	0.99994	1.02	0.00015	0.04709	1.739	0.00008	0.02413	1.774
ENSMUSG00000050721	Plekho2	0.00246	0.99994	1.413	0.02938	0.99994	1.311	<0.00001	0.00011	1.852
ENSMUSG00000030110	Ret	0.01198	0.99994	1.425	0.08464	0.99994	1.31	0.00003	0.00929	1.867
ENSMUSG00000029311	Hsd17b11	0.25149	0.99994	1.098	<0.00001	<0.00001	1.74	<0.00001	<0.00001	1.912
ENSMUSG00000028755	Cda	0.98695	0.99994	1.002	<0.00001	0.00058	1.913	<0.00001	0.00038	1.917
ENSMUSG00000023057	Fabp2	0.01448	0.99994	1.258	0.00001	0.00263	1.53	<0.00001	<0.00001	1.925
ENSMUSG00000002289	Angptl4	0.2168	0.99994	1.268	0.02817	0.99994	1.542	0.00062	0.10869	1.955
ENSMUSG00000034731	Dgkh	0.47949	0.99994	1.13	0.00486	0.67534	1.739	0.00047	0.08591	1.966
ENSMUSG00000000876	Pxmp4	0.31296	0.99994	1.119	<0.00001	0.00169	1.798	<0.00001	0.00001	2.012
ENSMUSG00000056666	Retsat	0.79361	0.99994	1.031	<0.00001	0.00001	2.056	<0.00001	<0.00001	2.119
ENSMUSG00000050097	Ces2b	0.28053	0.99994	-1.245	<0.00001	0.00186	2.798	0.00035	0.06804	2.247
ENSMUSG00000017697	Ada	0.02667	0.99994	1.733	0.19553	0.99994	1.383	0.00045	0.08475	2.398
ENSMUSG00000093805	Gal3st2b	0.12648	0.99994	1.187	<0.00001	0.00002	2.053	<0.00001	<0.00001	2.437
ENSMUSG00000026639	Lamb3	0.07329	0.99994	1.269	<0.00001	0.00038	1.978	<0.00001	<0.00001	2.51
ENSMUSG00000067231	Cyp2c65	0.36812	0.99994	1.11	<0.00001	<0.00001	2.275	<0.00001	<0.00001	2.525
ENSMUSG00000013653	1810065E05Rik	<0.00001	0.00162	1.634	<0.00001	0.00043	1.733	<0.00001	<0.00001	2.832
ENSMUSG00000030643	Rab30	0.53865	0.99994	1.127	0.00004	0.01275	2.587	<0.00001	0.001	2.917
ENSMUSG00000042010	Acacb	0.20294	0.99994	1.235	<0.00001	0.00149	2.447	<0.00001	<0.00001	3.022
ENSMUSG00000030329	Pianp	0.6487	0.99994	1.089	<0.00001	0.00043	3.556	<0.00001	0.00004	3.873

ENSMUSG00000031089	Slc6a14	0.00016	0.30066	9.159	0.22469	0.99994	-2.064	0.00929	0.73132	4.438
ENSMUSG00000033579	Fa2h	0.00707	0.99994	2.606	0.19498	0.99994	1.705	0.00011	0.0296	4.442
ENSMUSG00000010651	Acaa1b	0.32357	0.99994	1.219	<0.00001	<0.00001	4.385	<0.00001	<0.00001	5.347
ENSMUSG00000021620	Acot12	0.54935	0.99994	1.168	0.00007	0.02337	4.887	0.00001	0.00377	5.71
ENSMUSG00000022383	Ppara	0.00039	0.38141	1.513	<0.00001	<0.00001	3.817	<0.00001	<0.00001	5.775
ENSMUSG00000079299	Klrb1	0.54483	0.99994	-1.244	0.00021	0.06301	7.528	0.00106	0.16664	6.054
ENSMUSG00000067229	Cyp2c66	0.55838	0.99994	1.149	<0.00001	0.00002	7.814	<0.00001	<0.00001	8.977
ENSMUSG00000027875	Hmgcs2	0.05304	0.99994	1.427	<0.00001	<0.00001	7.69	<0.00001	<0.00001	10.974
ENSMUSG00000028435	Aqp3	0.07131	0.99994	1.411	<0.00001	<0.00001	9.523	<0.00001	<0.00001	13.437
ENSMUSG00000019577	Pdk4	0.13432	0.99994	1.376	<0.00001	<0.00001	9.84	<0.00001	<0.00001	13.539
ENSMUSG00000054422	Fabp1	0.00703	0.99994	1.401	<0.00001	<0.00001	10.961	<0.00001	<0.00001	15.353
ENSMUSG00000019768	Esr1	0.4765	0.99994	-1.066	<0.00001	<0.00001	17.619	<0.00001	<0.00001	16.523

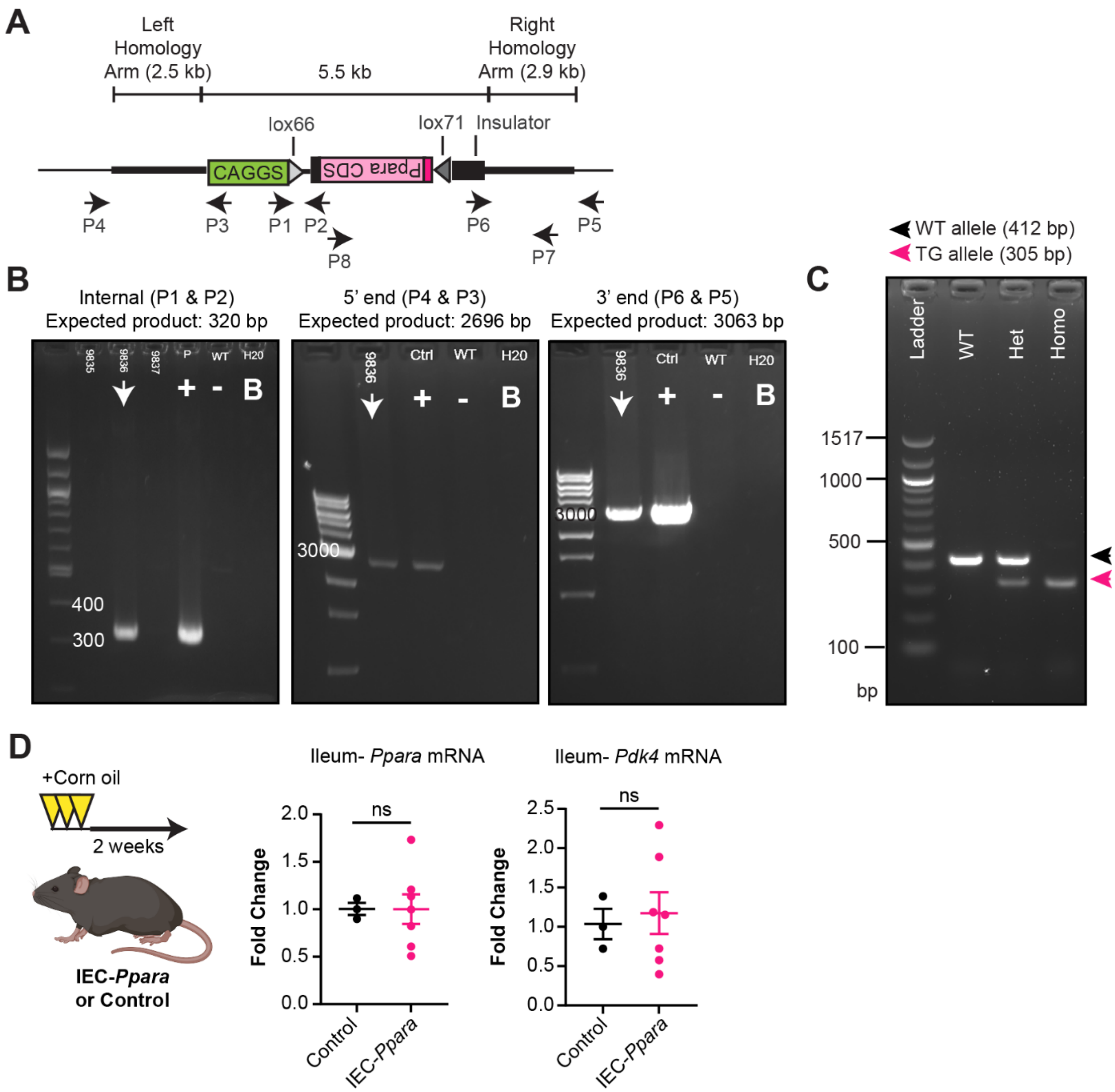

Supplementary Figure S1. Related to Figure 1: Validation of CAG-*Ppara*,-*EGFP* strain. (**A**) Schematic of the CAG-*Ppara*,-*EGFP* transgene cassette and the binding locations of the oligos (P1-P8) used to verify correct transgene insertion and orientation within *Col1a1* 3'UTR safe harbor site. (**B**) Representative agarose gel images showing PCR amplicons of the transgene internal region (left), 5' end (middle), and 3' end (right). Clone #9836 (arrows) presented with the expected PCR product sizes in base pairs (bp). +, positive control. -, negative control. B, water blank. A sanger sequencing reaction using primers P7 and P8 additionally supported correct transgene

orientation in clone #9836 (data not shown), which was subsequently used to establish the novel CAG-*Ppara*,-*EGFP* mouse strain. (**C**) Representative agarose gel image of PCR genotyping results for control, IEC-*Ppara* heterozygous, and IEC-*Ppara* homozygous mice. (**D**) IEC-*Ppara* homozygous (n=7) and control (n=3) mice were administered corn oil vehicle alone daily for 3 days and assessed two weeks later. *Ppara* and *Pdk4* gene expression were determined in ileal tissue by qRT-PCR. ns, not significant by unpaired t-test.

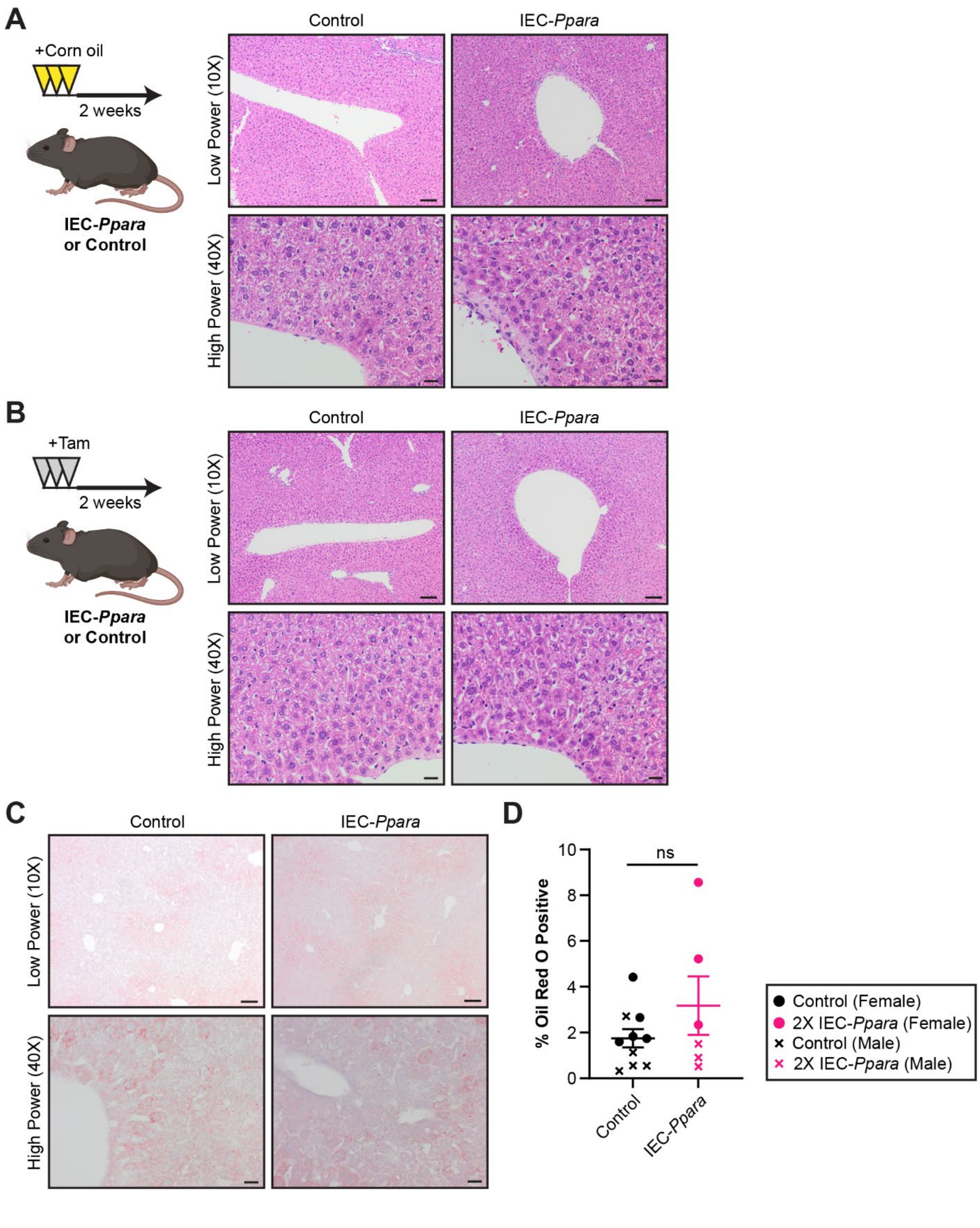

Figure S2. Related to Figure 3: Liver histology in IEC-*Ppara* mice. Mice were administered corn oil vehicle alone (n=3-7) (**A**) or tamoxifen (n=6-10) (**B**) daily for 3 days and assessed 2 weeks

later. (**A, B**) Representative microscopy images of H&E-stained liver tissue sections from the indicated genotypes showing low-power (scale bar, 100 μm) and high-power (scale bar, 20 μm) views of liver histology. (**C**) Representative microscopy images of liver tissue sections stained with Oil Red O. (**D**) Quantification of Oil Red O-positive area (n=6-10). ns, not significant as determined by unpaired t-test.

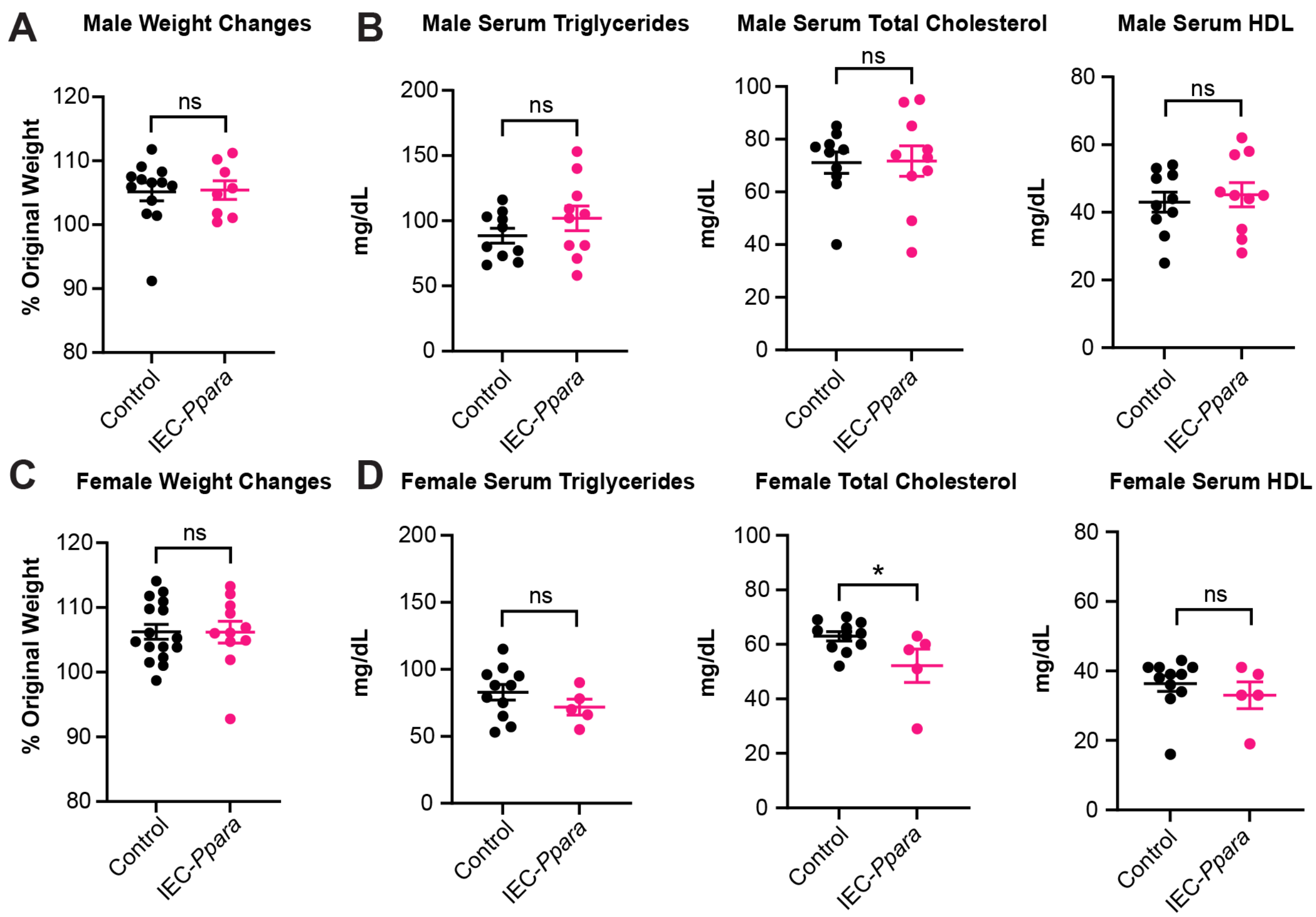

Figure S3. Related to Figure 3: Body weight and serum lipid panel by sex. (**A, C**) Quantification of body weight of control and IEC-*Ppara* mice relative to their original body weights shown separately for males (n=8-13) **(A)** and females (n=11-16) **(C)**. (**B, D**) Serum triglycerides (left), total cholesterol (middle), and HDL (right) shown separately for males (n=10) **(B)** and females (n=5-11) **(D)**. **P*<0.05, or ns, not significant as determined by unpaired t-test.

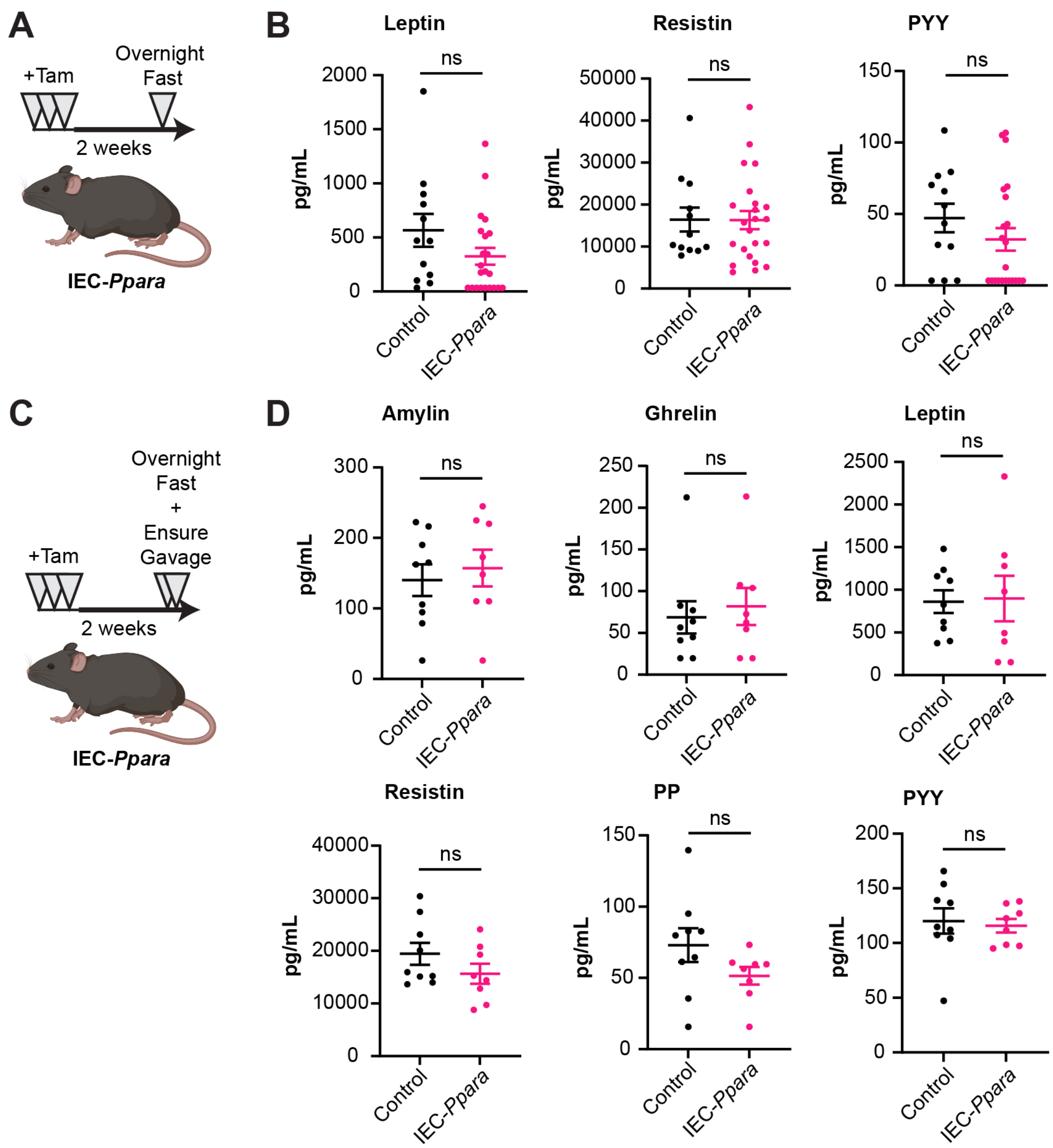

Figure S4. Related to Figure 5: Additional analysis of gut hormones. (**A**) Schematic of experiment design to collect serum analyzed in (B). (**B**) Quantification of serum leptin, resistin, and PYY (n=12-23). (**C**) Schematic of experiment design to collect serum analyzed in (D). Serum was collected 30 minutes after Ensure administration. (**D**) Quantification of serum amylin, ghrelin, leptin, resistin, PP, and PYY (n=8-9). ns, not significant as determined by unpaired t-test.

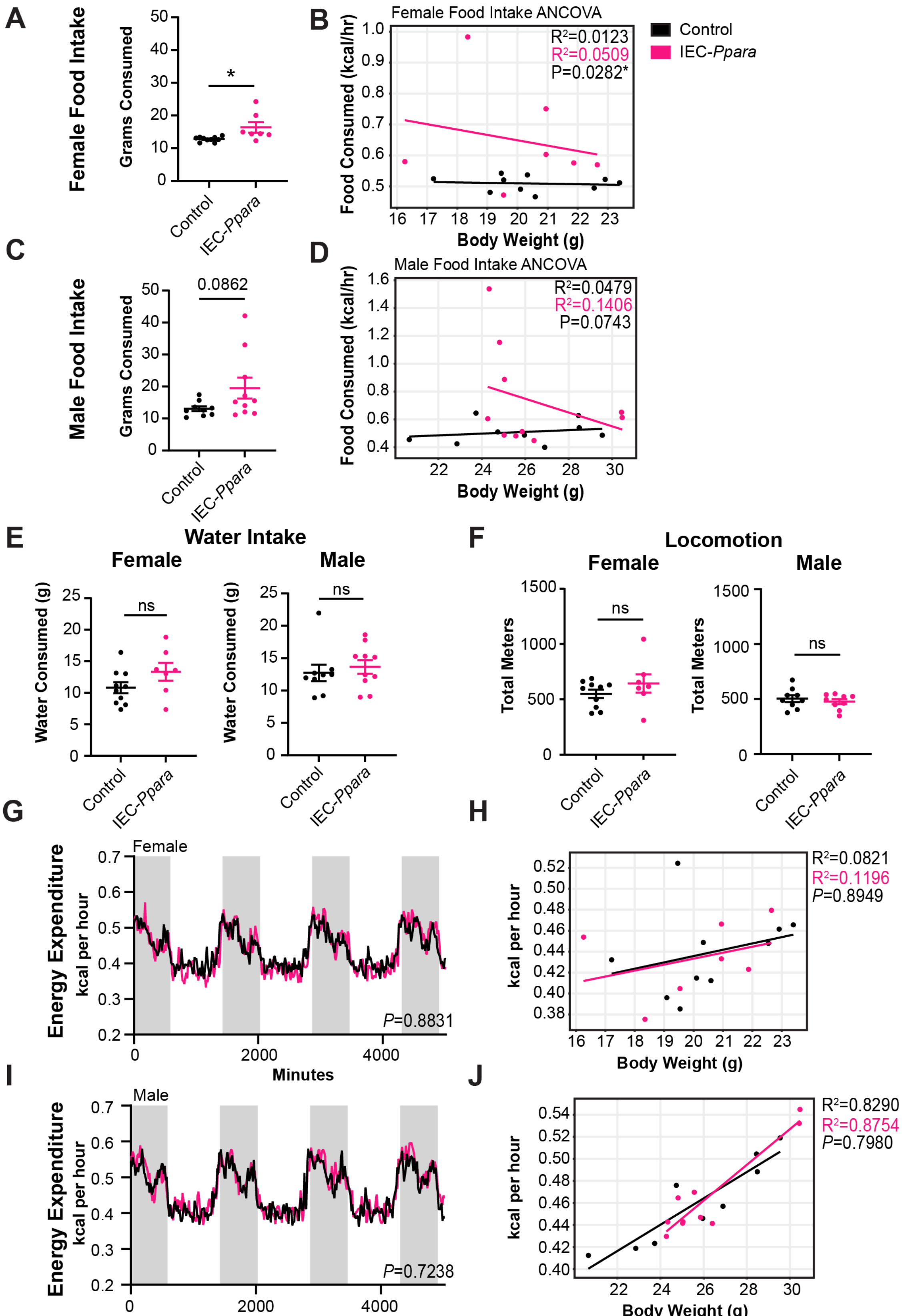
A
Female Food Intake
Grams Consumed
50
40
30
20
10
0
*
Control
IEC-Ppara
B
Female Food Intake ANCOVA
Food Consumed (kcal/hr)
1.0
0.9
0.8
0.7
0.6
0.5
R²=0.0123
R²=0.0509
P=0.0282*
16 17 18 19 20 21 22 23
Body Weight (g)
Control
IEC-Ppara
C
Male Food Intake
Grams Consumed
0.0862
Control
IEC-Ppara
D
Male Food Intake ANCOVA
Food Consumed (kcal/hr)
1.6
1.4
1.2
1.0
0.8
0.6
0.4
R²=0.0479
R²=0.1406
P=0.0743
22 24 26 28 30
Body Weight (g)
E
Water Intake
Female
Male
Water Consumed (g)
25
20
15
10
5
0
ns
Control
IEC-Ppara
F
Locomotion
Female
Male
Total Meters
1500
1000
500
0
ns
Control
IEC-Ppara
G
Energy Expenditure
kcal per hour
Female
0.7
0.6
0.5
0.4
0.3
0.2
P=0.8831
0 2000 4000
Minutes
H
kcal per hour
0.52
0.50
0.48
0.46
0.44
0.42
0.40
0.38
R²=0.0821
R²=0.1196
P=0.8949
16 17 18 19 20 21 22 23
Body Weight (g)
I
Energy Expenditure
kcal per hour
Male
P=0.7238
0 2000 4000
Minutes
J
kcal per hour
0.54
0.52
0.50
0.48
0.46
0.44
0.42
0.40
R²=0.8290
R²=0.8754
P=0.7980
22 24 26 28 30
Body Weight (g)

Figure S5. Related to Figure 6. Food intake, energy expenditure, locomotion, and water intake by sex. Mice were singly housed in metabolic cages and analyzed over an 83.5-hour period. Data are separated by sex, with data from females (n=7-10) in (A, B, E [left], F [left], G and H) and from males (n=9-10) in (C, D, E [right], F [right], I and J). **(A-D)** Food intake graphed for individual mice **(A, C)** and as an X-Y plot of ANCOVA using body weight as a covariate (**B, D**). **(E)** Cumulative water intake. **(F)** Directed locomotion. **(G-J)** Energy expenditure over time (**G, I**) and as X-Y plot of ANCOVA using body weight as a covariate (**H, J**). For B, D, H, and J, the exact *P*-value determined by ANCOVA and the R^2 values for control (black text) and IEC-*Ppara* (pink text) are shown. For G and I, the exact *P*-value determined by 2-way repeated measures ANOVA is shown. For A, C, E, and F, *$P<0.05$ or ns, not significant, as determined by unpaired t-test.

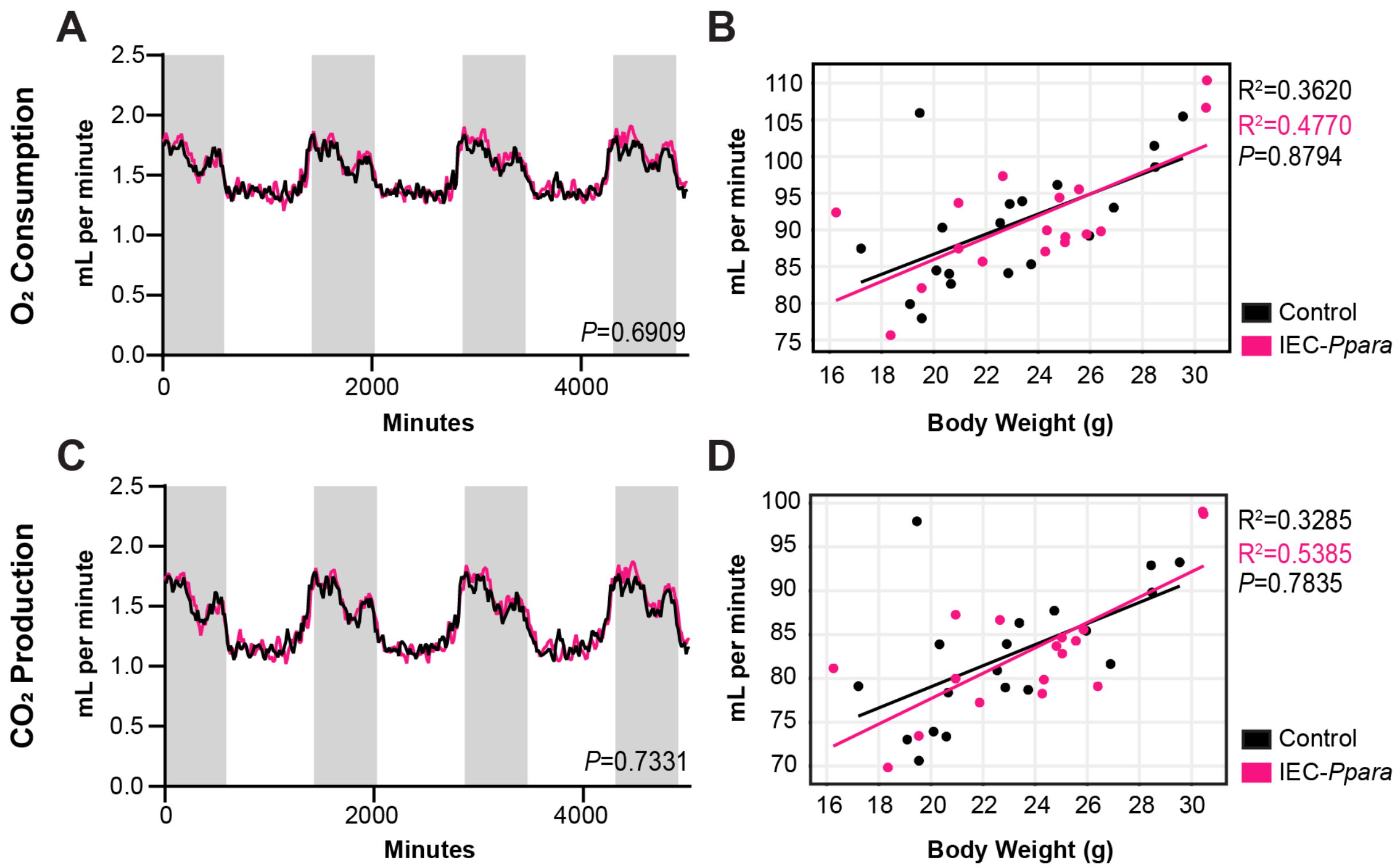

Figure S6. Related to Figure 6: **O_2 production and CO_2 consumption in IEC-*Ppara* mice.** (**A-D**) Mice were singly housed in metabolic cages and analyzed over an 83.5-hour period (n=16-19). Oxygen consumption over time (**A**) and as X-Y plot of ANCOVA using body weight as a covariate (**B**). Carbon dioxide production over time (**C**) and as X-Y plot of ANCOVA using body weight as a covariate (**D**). For A and C, the exact *P*-value determined by 2-way repeated measures ANOVA is shown. Dark photoperiod is represented by gray rectangles. For B and D, the exact *P*-value determined by ANCOVA and the R^2 values for control (black text) and IEC-*Ppara* (pink text) are shown.

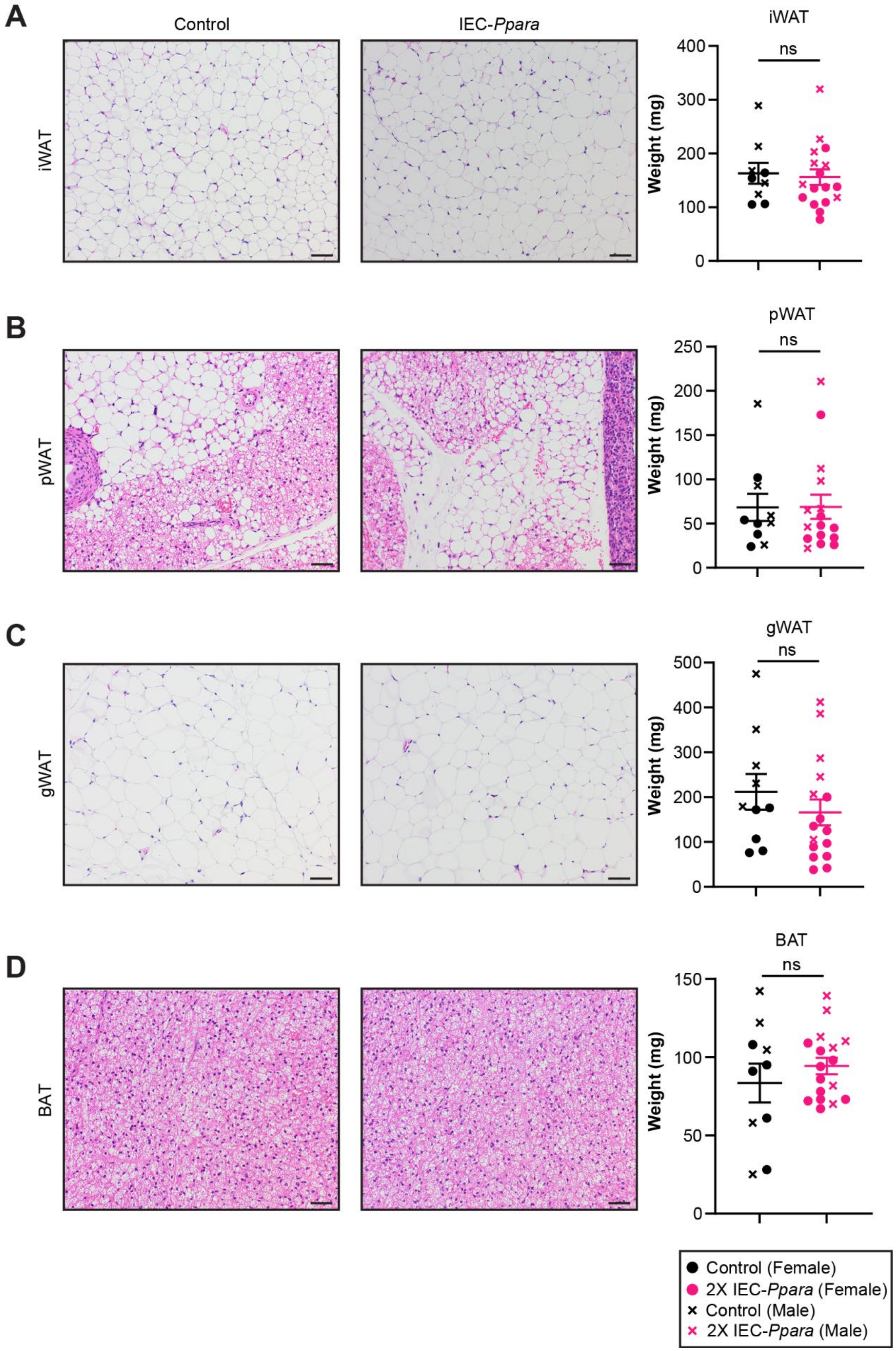
A
Control
IEC-*Ppara*
iWAT
iWAT
ns
Weight (mg)
400
300
200
100
0
B
pWAT
pWAT
ns
Weight (mg)
250
200
150
100
50
0
C
gWAT
gWAT
ns
Weight (mg)
500
400
300
200
100
0
D
BAT
BAT
ns
Weight (mg)
150
100
50
0
Control (Female)
2X IEC-*Ppara* (Female)
Control (Male)
2X IEC-*Ppara* (Male)

Figure S7 Related to Figure 6: Analysis of fat pads in IEC-*Ppara* mice. (**A-D**) Representative images of indicated fat pads in fasted control and IEC-*Ppara* mice (left). Scale bars, 50 μm. Inguinal white adipose tissue (iWAT); perirenal WAT (pWAT); gonadal WAT (gWAT); brown adipose tissue (BAT). Quantification of indicated fat pad weights (n=9-17) (right). ns, not significant as determined by t-test.
